# In vivo genome editing in mouse restores dystrophin expression in Duchenne muscular dystrophy patient muscle fibers

**DOI:** 10.1186/s13073-021-00876-0

**Published:** 2021-04-12

**Authors:** Menglong Chen, Hui Shi, Shixue Gou, Xiaomin Wang, Lei Li, Qin Jin, Han Wu, Huili Zhang, Yaqin Li, Liang Wang, Huan Li, Jinfu Lin, Wenjing Guo, Zhiwu Jiang, Xiaoyu Yang, Anding Xu, Yuling Zhu, Cheng Zhang, Liangxue Lai, Xiaoping Li

**Affiliations:** 1 Department of Neurology, The First Affiliated Hospital, Sun Yat-sen University; Guangdong Provincial Key Laboratory of Diagnosis and Treatment of Major Neurological Diseases, National Key Clinical Department and Key Discipline of Neurology; Zhongshan Medical School, Sun Yat-sen University; Center for Stem Cell Biology and Tissue Engineering, Key Laboratory for Stem Cells and Tissue Engineering, Ministry of Education, Guangzhou, 510080 China; 2 CAS Key Laboratory of Regenerative Biology, Guangdong Provincial Key Laboratory of Stem Cell and Regenerative Medicine, Guangzhou Institutes of Biomedicine and Health, Chinese Academy of Sciences, Guangzhou, 510530 China; 3 University of Chinese Academy of Sciences, Beijing, 100049 China; 4 Department of Neurology, Guangzhou First People’s Hospital, School of Medicine, South China University of Technology, Guangzhou, 510180 Guangdong China; 5 Department of Neurology, Seventh Affiliated Hospital of Sun Yat-sen University, Shenzhen, 518017 Guangdong China; 6 Scientific Instruments Centre, Guangzhou Institutes of Biomedicine and Health, Chinese Academy of Sciences, Guangzhou, 510530 Guangdong China; 7 Institute of Physical Science and Information Technology, Anhui University, Hefei, 230601 Anhui China; 8 Department of Neurology and Stroke Centre, The First Affiliated Hospital, Jinan University, Guangzhou, 510630 Guangdong China; 9 Bioland Laboratory (Guangzhou Regenerative Medicine and Health Guangdong Laboratory, GRMH-GDL), Guangzhou, 510005 China; 10 Research Unit of Generation of Large Animal Disease Models, Chinese Academy of Medical Sciences (2019RU015), Guangzhou, 510530 China; 11 Institute for Stem Cell and Regeneration, Chinese Academy of Sciences, Beijing, 100101 China; 12 Current address: Zhongshan Medical School, Sun Yat-sen University, No.72 Zhongshan Road 2, Guangzhou, 510080 China

**Keywords:** Duchenne muscular dystrophy, Gene editing, CRISPR/Cas9, CRISPR/ Cas12a, Patient-derived xenograft model

## Abstract

**Background:**

Mutations in the *DMD* gene encoding dystrophin—a critical structural element in muscle cells—cause Duchenne muscular dystrophy (DMD), which is the most common fatal genetic disease. Clustered regularly interspaced short palindromic repeat (CRISPR)-mediated gene editing is a promising strategy for permanently curing DMD.

**Methods:**

In this study, we developed a novel strategy for reframing *DMD* mutations via CRISPR-mediated large-scale excision of exons 46–54. We compared this approach with other DMD rescue strategies by using DMD patient-derived primary muscle-derived stem cells (DMD-MDSCs). Furthermore, a patient-derived xenograft (PDX) DMD mouse model was established by transplanting DMD-MDSCs into immunodeficient mice. CRISPR gene editing components were intramuscularly delivered into the mouse model by adeno-associated virus vectors.

**Results:**

Results demonstrated that the large-scale excision of mutant *DMD* exons showed high efficiency in restoring dystrophin protein expression. We also confirmed that CRISPR from *Prevotella* and *Francisella* 1(Cas12a)-mediated genome editing could correct *DMD* mutation with the same efficiency as CRISPR-associated protein 9 (Cas9). In addition, more than 10% human DMD muscle fibers expressed dystrophin in the PDX DMD mouse model after treated by the large-scale excision strategies. The restored dystrophin in vivo was functional as demonstrated by the expression of the dystrophin glycoprotein complex member β-dystroglycan.

**Conclusions:**

We demonstrated that the clinically relevant CRISPR/Cas9 could restore dystrophin in human muscle cells in vivo in the PDX DMD mouse model. This study demonstrated an approach for the application of gene therapy to other genetic diseases.

**Supplementary Information:**

The online version contains supplementary material available at 10.1186/s13073-021-00876-0.

## Background

Duchenne muscular dystrophy (DMD) is an X-linked recessive hereditary disease and the most frequent lethal inherited disease, affecting 1 in about 3500–5000 newborn males [1]. Mutations in the dystrophin gene (*DMD*) that disrupt the reading frame and lead to prematurely aborted dystrophin synthesis give rise to DMD. Dystrophin is a critical cytoskeletal structural protein in muscle cells that stabilizes the dystrophin glycoprotein complex (DGC) at the sarcolemma [2]. Loss of functional dystrophin leads to DGC degradation and muscle fiber membrane fragility, resulting in progressive muscle degeneration [3] that leaves patients wheelchair-bound at about the age of 12 [4] and premature death as they reach their twenties. To date, no effective treatments for DMD have been developed.

*DMD* is the largest known gene with 2.6 million base pairs (bp) comprising 79 exons. Its large size makes it prone to mutations. In-frame *DMD* mutations generate an internally truncated but partly functional dystrophin protein that results in Becker muscular dystrophy (BMD), which is observed in 1 in about 20,000 male births and has an intermediate to mild clinical manifestation, with some cases being asymptomatic into the seventh decade of life [5]. Efforts have been made to rescue dystrophin expression in DMD by overexpressing a truncated but functional micro-dystrophin-utilizing lentivirus or adeno-associated virus (AAV) [6–8] or by inducing the skipping of out-of-frame exons with antisense oligonucleotides [9–11] and the read-through of nonsense mutations with small molecules [12]. However, these approaches are constrained by the virus packaging limit and by the failure to permanently restore dystrophin expression due to the lack of genome manipulation.

Clustered regularly interspaced short palindromic repeats (CRISPR) genome editing technology shows promise for the treatment of genetic disorders [13–19]. Precise genome modifications to correct mutations in genomic DNA can be achieved by CRISPR-associated protein 9 (Cas9) - or CRISPR -associated protein 12a (Cas12a)-mediated cleavage of DNA sequences targeted by a single guide RNA (gRNA). Inspired by BMD with in-frame mutant *DMD*, recent studies have demonstrated that CRISPR/Cas9- or Cas12a-mediated removal of one or more exons to reframe mutant *DMD* restores dystrophin expression in cultured cells from DMD patients and in animal models of the disease [20–27]. Reframing mutant *DMD* by deleting exons 45–55, which can correct about 60–70% of human DMD mutations, is a commonly used strategy [20, 21, 28]. Although therapies based on CRISPR-Cas9 genome editing are efficient, their immunology and efficiency in human cells in vivo must be comprehensively examined before experiments could be conducted in humans. Preexisting immunity, including an innate and adaptive cellular immune response to Cas9 [29], and the presence of anti-Cas9 antibodies [30] and T cells [31], reportedly decreases the efficacy of CRISPR systems and may cause serious safety problems. Whether CRISPR systems could work in human muscle fibers in vivo is another critical issue that must be clarified for the clinical treatment of DMD.

In this study, using immunodeficient NOD-scid-IL2Rg^−/−^ (NSI) mice and DMD patient muscle-derived stem cells (DMD–MDSCs), we developed a patient-derived xenograft (PDX) DMD mouse model to conduct in vivo genome editing via AAV-mediated CRISPR system and restore the expression of dystrophin in DMD patient muscle fibers. We also designed new correction strategies that involve the deletion of exons 46–54 and the application of Cas12a with comparable restoration efficiency. This work might help in realizing the promise of CRISPR-mediated gene therapy for DMD treatment.

## Methods

### Plasmid constructs

Expression cassettes for human codon-optimized Cas9 and Cas12a nuclease and a panel of gRNAs were designed and constructed as previously described [32, 33]. The dual fluorescence reporter vector was constructed on the basis of the pCMV-tdTomato vector. In brief, we added the EGFP coding sequence and an internal ribosome entry site (IRES) into the pCMV-tdTomato vector to obtain the intermediate vector pCMV–EGFP–IRES–TdTomato. The cassette harboring introns 45 and 54 with targeting sites flanking a multi-polyA sequence was inserted into pCMV–EGFP–IRES–TdTomato downstream of EGFP and upstream of IRES.

### Isolation of MDSCs

Gastrocnemius muscle samples obtained from a 4-year-old male with DMD and a healthy control subject were immersed in gentamicin (Gibco, Grand Island, NY, USA) diluent (gentamicin: high-glucose Dulbecco’s Modified Eagle’s Medium [DMEM] (m/v) = 1 g:40 mL) for 15 min and then transferred onto a clean surface for the subsequent steps. The samples were washed three times in phosphate-buffered saline (PBS) supplemented with 1% penicillin–streptomycin (Gibco). Fascia, connective tissues, fat, and blood vessels were removed from the muscle tissues and then minced using forceps and surgical scissors. A coarse slurry was obtained, which was enzymatically digested at 37 °C in three volumes of an isopyknic mixture of 0.2% neutral protease (Roche Diagnostics, Mannheim, Switzerland) and 0.1% collagenase II (Gibco) for 1 h. The slurry was then centrifuged at 1000 rpm for 3 min. The supernatant was discarded, and the cell pellet was enzymatically digested at 37 °C by isopyknic 0.1% trypsin (Gibco) for 30 min and then centrifuged as described above. The cell pellet was passed through a 0.45-μm filter (Millipore, Billerica, MA, USA). Dissociated cells were seeded in a tissue culture flask coated with collagen type I (Roche Diagnostics) and cultured under standard culture conditions (37 °C, 5% CO_2_, and 95% relative humidity).

MDSCs were isolated via a modified version of the pre-plate technique as previously described [34]. In brief, 2 h after seeding the cells in the flask (pre-plate [PP]1), nonadherent cells and the medium were collected again and transferred into a fresh flask 24 h later (PP2). Serial pre-plating was performed to separate cells until PP6. The adherent cells at PP6 were deemed as MDSCs; these were cultured until they reached 80% confluence and then passaged at a ratio of 1:3. The DMD patient and the healthy control subject were recruited at The First Affiliated Hospital of Sun Yat-sen University.

### Cell culture, differentiation, and transfection

HEK293T cells were maintained in DMEM supplemented with 10% fetal bovine serum (FBS) and 1% penicillin–streptomycin. The MDSCs were maintained in DMEM supplemented with 10% horse serum, 10% FBS, 1% penicillin–streptomycin, and 0.5% chick embryo extract. The MDSCs were differentiated into myotubes by replacing the growth medium with DMEM supplemented with 2% heat-inactivated horse serum for 7 days. All reagents were purchased from Gibco. The cells were maintained at 37 °C and 5% CO_2_ and then transfected by electroporation by using the Neon Transfection kit (Thermo Fisher Scientific, Waltham, MA, USA).

### Transplantation of DMD-MDSCs into NSI mice

NSI mice were maintained under specific pathogen-free conditions. Bilateral tibialis anterior (TA) muscles of 6- to 8-month-old mice were locally pretreated with cardiotoxin (10 μL, 10 μM/50 μL, Sigma-Aldrich, St. Louis, MO, USA) 24 h prior to transplantation to stimulate the migration of engrafted cells and the formation of new myofibers. After the mice were anesthetized by abdominal injection of 5% chloral hydrate, the TA muscle was exposed, and 1 × 10^6^ DMD–MDSCs resuspended in 10 μL Matrigel (Corning Inc., Corning, NY, USA) supplemented with 0.1 μg human basic fibroblast growth factor (R&D Systems, Minneapolis, MN, USA) were slowly injected at an oblique angle into five to seven sites in the muscle by using a 10 μL 33G microsyringe (Hamilton Co., Reno, NV, USA). The skin was then sutured closed. NSI mice injected with Matrigel served as the negative control. TA muscles were harvested 30 days post transplantation for immunohistochemical analysis.

### Bioluminescence imaging

A cooled CCD camera system (IVIS 100 Series Imaging System, Xenogen, Alameda, CA, USA) was used for imaging as previously described. Images were acquired following intraperitoneal injection of D-Luciferin in 250 μL of DPBS (15 mg/mL). Quantification was performed using Living Image software (Xenogen).

### AAV production and injection

CRISPR AAV vectors were generated by PackGene Biotech Co. (Guangzhou, China). For intramuscular injection into NSI mice, the animals were anesthetized by abdominal injection of 5% chloral hydrate, followed by injection of CRISPR AAV vector (25 μL, 1.5E + 12 vg) into the TA muscle transplanted with DMD–MDSCs.

### Analysis of off-target effects

The criterion for screening potential off-target sites was one to three mismatches with gRNAs. The website https://crispr.bme.gatech.edu [35] was used for the prediction of off-target sites for Cas9, and http://www.rgenome.net/cas-offinder/ [36] was used for the prediction of off-target sites for Cas12a. Primers corresponding to these sites were designed for PCR amplification of DNA fragments, with the genomic DNA extracted from gRNA- and Cas9/Cas12a-transfected HEK293 cells used as the template. PCR products were no more than 200 bp and used for library construction. High-throughput sequencing was performed with a mixture of equal amounts of PCR amplicons on an Illumina MiSeq System (San Diego, CA, USA).

### Whole-exome sequencing

Genomic DNA was extracted from the myotubes differentiated from WT, CRISPR-edited, and negative controls by using the Cell Genome Extraction kit (Tiangen Biotech, Beijing, China). The coding exons and untranslated region were analyzed via whole-exome sequencing. Quality control was performed by removing adapter sequences and reads with low complexity or of low quality by using Trimmoatic (version 0.39). Afterward, BWA (version 0.7.17-r1188) was used to align clean reads to hg38 human genome downloaded from UCSC. Samtools (version 1.9) was used to sort and index bam files, and Picard (version 2.20.5) was applied for marking duplicates. GATK (version 4.1.3.0) was used for the subsequent steps: (1) base quality score recalibration, (2) variant discovery, and (3) variant quality score recalibration. Known sites resource (e.g., SNPs from 1000G Project) was used for the variant quality score recalibration (VQSR) procedure with the parameters (-an MQ -an MQRankSum -an ReadPosRankSum -an FS -an SOR), and tranche sensitivity threshold was set to 90. The variants that passed the VQSR procedure were considered to be true variants. Variant annotation and further filtration were conducted using ANNOVAR (version 2019Oct24) and whole-genome databases (exac03, avsnp150). Variants annotated to known sites were eliminated. During the downstream analysis, the variants that simultaneously appeared in the treated, untreated, and sham-treated (PBS, Cas9) groups were considered as background noise and thus eliminated in further analysis. Genomic tracks of single-nucleotide variants (SNVs) and indels were drawn using Circlize (version 0.4.4), and ggplot2 (3.3.0) was used for other customized visualizations.

### PCR analysis

End-point PCR was performed with the primers shown in Additional file 1: Figure S1A to detect the deletion of exons 46–54 in HEK293 cells when screening gRNAs. The loci of exon 51, exons 45–55, exons 46–54, and exon 50 were amplified from genomic DNA via PCR by using the primers flanking each locus to determine whether exon deletions or indels occurred in the MDSCs after genome editing both in vitro and in vivo. Afterward, the PCR product of INDEL50 was digested using T7E1. In vivo genome editing of the loci of exons 45–55 and exons 46–54 were amplified by 45 cycles. All PCR products were separated on 1.2% agarose gel and stained with ethidium bromide for analysis. The bands were cloned and sequenced to confirm the presence of expected intron junctions.

### RNA extraction and RT-PCR analysis

WT, CRISPR-targeted, and untargeted MDSCs were differentiated into myotubes. The cells were trypsinized and collected, and total mRNA was extracted using TRIzol reagent (Life Technologies) and reverse-transcribed into cDNA by using the FastQuant RT kit (Tiangen Biotech). PCR primers were designed to anneal to exons 44 and 56 to detect exon 45–55 deletion (∆45–55), exons 45 and 55 to detect exon 46–54 deletion (∆46–54), or exons 49 and 52 to detect indels in exon 50 (INDEL50). The PCR products were separated on 1.2% agarose gel and stained with ethidium bromide. The resolved bands were cloned and sequenced to verify the presence of expected exon junctions.

### qPCR for detection of human cells in PDX DMD mouse

Genomic DNA was extracted from the TA muscle of PDX DMD mouse. qPCR assay was conducted according to the protocol of Cohen et al. [37].

### Western blotting

For Western blotting of dystrophin protein, the MDSCs were differentiated into myotubes. The cells were lysed in radioimmunoprecipitation buffer (Beyotime Institute of Biotechnology, Shanghai, China) supplemented with phenylmethylsulfonyl fluoride (1 mmol/L). Total protein was separated on 6% SDS–PAGE gel, transferred onto a polyvinylidene difluoride membrane (Millipore) that was blocked with 5% skim milk and probed with mouse anti-dystrophin antibody (1:100, MABT827; Millipore), and then incubated with horseradish peroxidase-conjugated secondary antibody. The protein band was visualized using Super ECL Plus (Applygen Technologies, Beijing, China) according to the manufacturer’s instructions.

### Immunofluorescence analysis

Myotubes differentiated from the MDSCs seeded on 12 mm coverslips were fixed with cold acetone for 10 min at − 20 °C. The coverslips were washed three times with PBS and blocked for 1 h with 5% bovine serum albumin (BSA)/PBS solution at room temperature, followed by overnight incubation at 4 °C in a primary antibody cocktail of rabbit anti-dystrophin (1:200, ab15277; Abcam, Cambridge, UK) and mouse anti-MHC (1:200, A4.1025; Developmental Studies Hybridoma Bank, Iowa City, IA, USA) antibodies in 3% BSA/PBS solution. The coverslips were washed three times with PBS and incubated for 1 h at room temperature with a secondary antibody cocktail containing Alexa Fluor 555-conjugated goat anti-rabbit (1:1000, # 4413S; Cell Signaling Technology, Danvers, MA, USA) and Alexa Fluor 488-conjugated goat anti-mouse (1:1000, #4408S; Cell Signaling Technology) antibodies. After three washes with PBS, the coverslips were inverted onto slides overlaid with DAPI mounting medium (Sigma-Aldrich) for nuclear counterstaining. The slides were visualized with a confocal microscope (Zeiss, Oberkochen, Germany). Ten coverslips were assigned for each strategy, WT, and untargeted MDSC. Ten images of each slide were captured to quantify dystrophin- and MHC-positive fibers and determine their ratio, which was compared for different gene-editing strategies.

TA muscles harvested from mice were frozen in isopentane precooled with liquid nitrogen, and serial cryosections were cut at a thickness of 8 μm. Samples from mice transplanted with DMD–MDSCs were labeled with a primary antibody cocktail containing rabbit anti-laminin (1:400, ab11575; Abcam), mouse anti-Lamin A+C (1:100, ab40567; Abcam), and mouse anti-spectrin (1:100, NCL-SPEC1; Novocastra, Newcastle upon Tyne, UK) antibodies and a secondary antibody cocktail containing Alexa Fluor 488-conjugated goat anti-rabbit (1:1000, #4412S; Cell Signaling Technology) and Alexa Fluor 555-conjugated goat anti-mouse (1:1000, #4409S; Cell Signaling Technology) antibodies. Samples from the CRISPR-targeted and nontargeted PDX DMD mice were labeled with a primary antibody cocktail containing rabbit anti-laminin (1:400, ab11575; Abcam), mouse anti-Lamin A+C (1:100, ab40567; Abcam), and mouse anti-dystrophin (1:50, MABT827; Millipore) antibodies and a secondary antibody cocktail containing Alexa Fluor 555-conjugated goat anti-rabbit (1:1000, #4413S; Cell Signaling Technology) and Alexa Fluor 488-conjugated goat anti-mouse (1:1000, #4408S; Cell Signaling Technology) antibodies. A total of 40 mice were tested, with 8 mice (4 males, 4 females) each strategy and for the control. Both TA muscles of the same mouse were treated in the same manner, and they were averaged to give one result per mouse. Ten images of each section were captured to quantify dystrophin-positive fibers and Lamin A+C-positive nuclei and determine their ratio, which was compared among different gene-editing strategies. Immunofluorescence images were analyzed blind to sample identity.

### RNA-seq

The TruSeq RNA Sample Preparation kit (Illumina) was used to construct RNA-seq libraries. High-throughput sequencing was performed on the HiSeq 2500 system (Illumina). Quality control was performed by removing adapter sequences and reads with low complexity or of low quality. Subsequently, Salmon (version 0.8.2) was used to align clean reads to hg38 human transcriptome downloaded from Ensembl. In the differential analysis part, to ensure that all the differential genes were generated by gene therapy, unedited, PBS, and Cas9 were used as controls for differential analysis, and then the intersections of their differential genes were taken as the true differential genes. Differential expression analysis was performed using DESeq2 (version 1.20.0), and genes with an adjusted *P* < 0.05 and fold change > 2 were considered differentially expressed. Gene Ontology analysis of the differentially expressed genes was performed using clusterProfiler (version 3.10.1).

### Quantitative droplet digital PCR (ddPCR)

ddPCR was performed on the edited genomic DNA samples by using a QX200TM Droplet DigitalTM PCR System with QX200TM ddPCRTM EvaGreen Supermix (BioRad). Four primer sets were designed, including primers specific for exon 57 (E57-F, E57-R), which detected intact dystrophin gene, and primers specific for deletion products (∆46–54/Cas9-F, ∆46–54/Cas9-R; ∆46–54/Cas12a-F, ∆46–54/Cas12a-R; ∆45–55/Cas9-F, ∆45–55/Cas9-R). The primer sequences are given in Additional file 2: Table S8.

### Nanopore sequencing analysis

The cells were trypsinized and collected, and genomic DNA was extracted using the Cell Genome Extraction kit (Tiangen Biotech, Beijing, China) for Oxford Nanopore sequencing library construction. High-throughput sequencing was then performed on MinION. Porechop (version 0.2.4) was used to remove adapter sequences from Nanopore reads. Minimap2 (version 2.17-r941) was utilized to map long reads to the human genome. Samtools (version 1.9) was employed to process bam files. bamCoverage (version 3.3.0) was used to convert bam files to bigwig files. Continuous genome reference sequences of three different strategies were also established by predicting the precise connection after editing, and then Nanopore reads were mapped to this reference sequence to explore change models and reads coverage around the cut sites. Reads coverage in bigwig files or bam files was visualized using the UCSC genome browser or the IGV genome browser. Density plot and mutation percentage of bases were counted by custom R scripts.

### Genome-wide, unbiased identification of DSBs enabled by sequencing (GUIDE-seq) analysis

The GeneRulor NGS Library Prep Kit was used to construct GUIDE-seq libraries. The guideseq package (https://github.com/aryeelab/guideseq) [38] was implemented to preprocess and analyze the GUIDE-Seq data. When mapping potential off-target sites, the cutoff for alignment to the on-target spacer sequence was set at 8 mismatches for 21 nucleotide spacers, 9 mismatches for 22 nucleotide spacers, and 10 mismatches for 23 nucleotide spacers. For all mapped reads, an RPKM value (reads number/sum of reads number × 1000) was calculated to filter out detected sites with lesser reads (RPKM < 1). The potential off-target sites detected were visualized on the genomic scales by using the R package karyoploteR (version 1.8.8).

### Statistical analysis

Data from continuous variables are expressed as mean ± SEM. The means of two groups were compared by Student’s *t* test, and *P* < 0.05 was considered statistically significant.

## Results

### Strategy for CRISPR/Cas9-mediated correction of the DMD gene

**Fig. 1:**
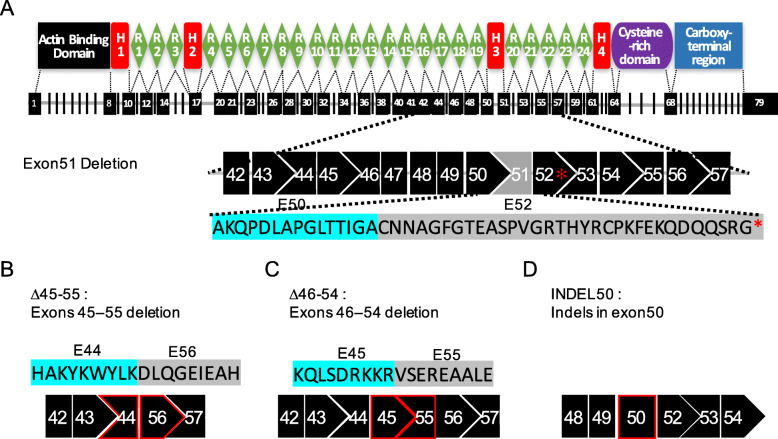
Structure of human dystrophin protein and strategies for targeting the mutant human *DMD* gene. **a** Schematic illustration of the human dystrophin protein structure and the reading frame of the *DMD* gene derived from a DMD patient harboring a deletion of exon 51 and a termination codon in exon 52 (red asterisk). H, Hinge; R, Repeat; E, Exon. **b** Strategy I: deletion of exons 45–55 (∆45–55) joins exon 44 to exon 56 and restores the reading frame. E: Exon. **c** Strategy II: deletion of exons 46–54 (∆46–54) joins exon 45 to exon 55 and restores the reading frame. E, exon. **d** Strategy III: introduction of random indels in exon 50 (INDEL50) has a chance of restoring the reading frame

The dystrophin protein is mainly composed of four domains, of which the N-terminal, the C-terminal, and the cysteine-rich domains are functionally more important and serve as binding sites of dystrophin and transmembrane protein or sarcolemma. By contrast, much of the central rod domain, which contains the mutational “hot spot,” is not functionally necessary (Fig. 1a). Genome editing to reframe DMD by CRISPR-mediated nonhomologous end joining has been demonstrated as an effective approach for rescuing the expression of functional truncated dystrophin, and it has been shown to result in conversion of DMD to a BMD-like phenotype. Efficiency, applicability, and safety are the major concerns in the clinical application of CRISPR-mediated genome editing. In this study, DMD–MDSCs with deletion of exon 51, which introduces a termination codon in exon 52 and disrupts the open reading frame (ORF) (Fig. 1a), were used as target cells to optimize the strategy for CRISPR/Cas9-mediated gene therapy of DMD. CRISPR gene editing restored the disrupted ORF by inducing large-scale deletion of multiple exons or by introducing insertion or deletion mutations (indels) in exon 50; correlative gRNAs were designed targeting introns 44, 45, 50, 54, and 55 (Additional file 2: Table S1). In the first strategy (∆45–55), exons 45–55 (708 kb; Fig. 1b), which accounted for approximately 63% of the mutations of the DMD gene, were deleted. In the second strategy (∆46–54), exons 46–54 were deleted (334 kb; Fig. 1c) to reframe the mutant DMD, thereby preserving more of the rod domain and making dystrophin with a potentially better function. In the third strategy (INDEL50), the 3′ end of exon 50 was targeted with a single gRNA to restore the downstream reading frame via introduction of indels (Fig. 1d). Compared with the strategies for large fragment deletion, introducing indels improved the efficiency of cutting the genome (only one gRNA).

### CRISPR/Cas9-mediated reframing of human mutant DMD gene

**Fig. 2:**
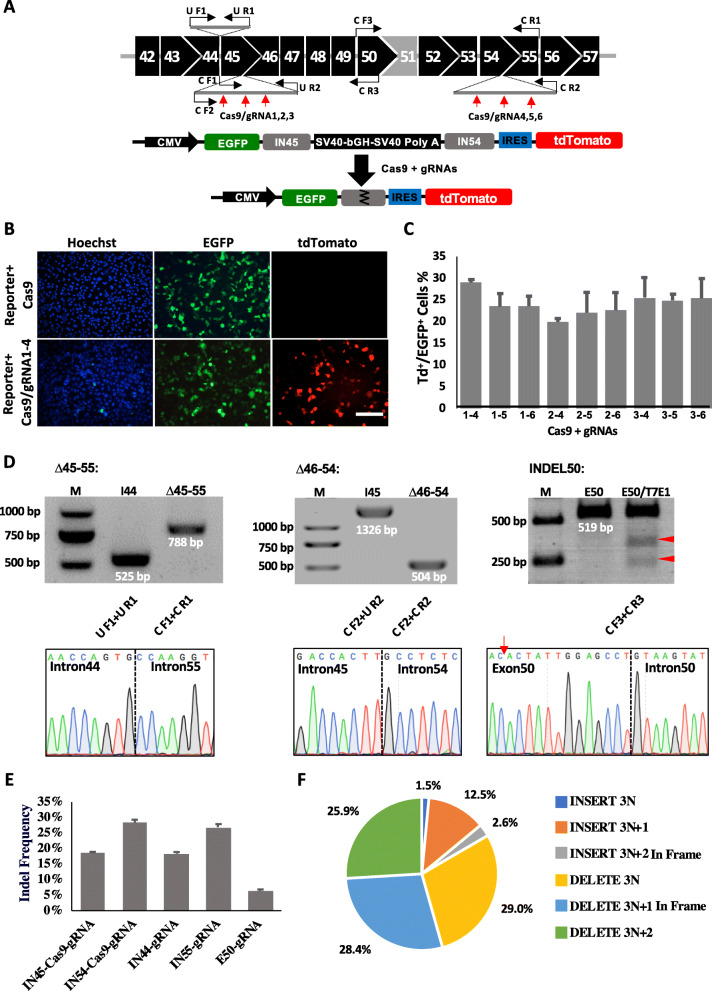
Screening gRNAs for CRISPR/Cas9-mediated targeting of the *DMD* gene and reframing of mutant *DMD* in DMD–MDSCs. **a** Schematic illustration of gRNAs targeting introns 45 and 54 of the *DMD* gene (top row). Red and black arrows indicate the target sites of gRNAs and the primer binding sites for the PCR experiments shown in **d**. Illustration of a dual-fluorescence reporter plasmid (bottom row). **b** HEK293 cells were co-transfected with the reporter construct and Cas9 (top row) or Cas9/gRNA1–4 (bottom row) by electroporation, and EGFP and tdTomato (Td) expression was observed with a fluorescence microscope 2 days later. Scale bar, 100 μm. **c** Flow cytometry analysis of HEK293 cells co-transfected with the reporter construct and Cas9/gRNA (*n* = 2). **d** Confirmation of the reframing of mutant *DMD* in DMD–MDSCs by PCR and Sanger sequencing; ∆45–55 yielded an intron 44/intron 55 junction (left column), ∆46–54 yielded an intron 45/intron 54 junction (middle column), and INDEL50 yielded T7E1-cleaved PCR amplicon fragments of the expected sizes (red arrowheads); the red arrow indicates the gRNA target site (right column). M, marker; I, intron; E, exon. **e** Targeting efficiency of gRNAs in DMD-MDSCs. **f** The ratio of different types of indels in INDEL50. The numbers of reads of different indel types in three replicates was averaged, and the ratio was calculated. INSERT 3 N+2 and DELETE 3 N+1 were in-frame mutations

gRNAs targeting introns 44 and 55 and exon 50 for the first (∆45–55) and third (INDEL50) strategies were designed on the basis of the screen for *DMD* targeting as previously reported [20, 21]. To achieve effective deletion of exons 46–54 (∆46–54; Fig. 1c), we generated a dual-fluorescence reporter to rapidly assess the genomic deletion efficiency of Cas9/gRNAs targeting introns 45 and 54. The reporter contained enhanced green fluorescent protein (EGFP) and tdTomato genes separated by *DMD* introns 45 and 54, which included the gRNA target sequence and flanked by a multi-polyA sequence with a robust transcription termination function (Fig. 2a; Additional file 2: Table S2). CRISPR/Cas9-mediated excision was predicted to remove the multi-polyA cassette to activate tdTomato expression. HEK293 cells were co-electroporated with gRNA targeting *DMD* intron 45 (gRNA nos. 1–3) and another targeting *DMD* intron 54 (gRNA nos. 4–6) along with Cas9 and the reporter construct (Additional file 1: Figure S1A). After 48 h, dual fluorescence was detected under a microscope (Fig. 2b), and excision efficiency was evaluated by flow cytometry (Fig. 2c; Additional file 1: Figure S2). Genomic end-point PCR was performed to detect excision of the *DMD* gene from genomic DNA in the transfected HEK293 cells. The expected genomic deletions were observed for most of the gRNA combinations (Additional file 1: Figure S1B). gRNAs 1–4 showed the highest gene-editing activity according to the results of fluorescence reporter, and these were used for genetic correction of *DMD* (Additional file 2: Table S3). Sanger sequencing confirmed the joining of introns 45 and 54 mediated by Cas9/gRNA1–4 (Additional file 1: Figure S1C). To test the specificity of Cas9/gRNA-mediated cleavage, we identified 24 potential off-target sites of gRNA1 and gRNA4 via an in silico prediction method and performed deep sequencing of HEK293 cells transfected with Cas9 and individual gRNA expression cassettes. Results showed that off-target mutagenesis occurred at a low frequency (Additional file 1: Figures S1D–S1F; Additional file 2: Table S4).

To reframe the mutant *DMD* gene in the DMD–MDSCs, the gRNAs for the three targeting strategies (Figs. 1b–d) were separately introduced along with Cas9 into the DMD–MDSCs by electroporation. After 3 days, genomic DNA was extracted from the transfected DMD–MDSCs for analysis. Genomic deletions and indel mutations were assessed by end-point PCR and the Surveyor nuclease assay. The expected deletion products of 708 kb and 334 kb in ∆45–55 and ∆46–54 targeted cells were detected via PCR of the genomic DNA. Sanger sequencing confirmed the reframing of mutant *DMD* (Fig. 2d; Additional file 1: Figures S3A and S3B). Notably, INDEL50 induced a conversion to three reading frames, with only 1/6 of the mutations restoring the ORF of mutant *DMD* as previously reported (Additional file 1: Figure S4). The targeting efficiency of gRNAs in the DMD–MDSCs was also detected by high-throughput sequencing of the PCR products of the edited *DMD* gene (Fig. 2e; Additional file 1: Figure S5; Additional file 2: Table S5). About 6% of the reads included indels from E50-gRNA-edited PCR products, in which 31% of the edited reads were in-framed (Fig. 2f). However, 1–2% of the reframed reads contained stop codons, and about 30% of the reframed reads lost the 5′ splice site (SS) (Additional file 2: Table S6).

### Restoration of dystrophin expression by CRISPR/Cas9

**Fig. 3:**
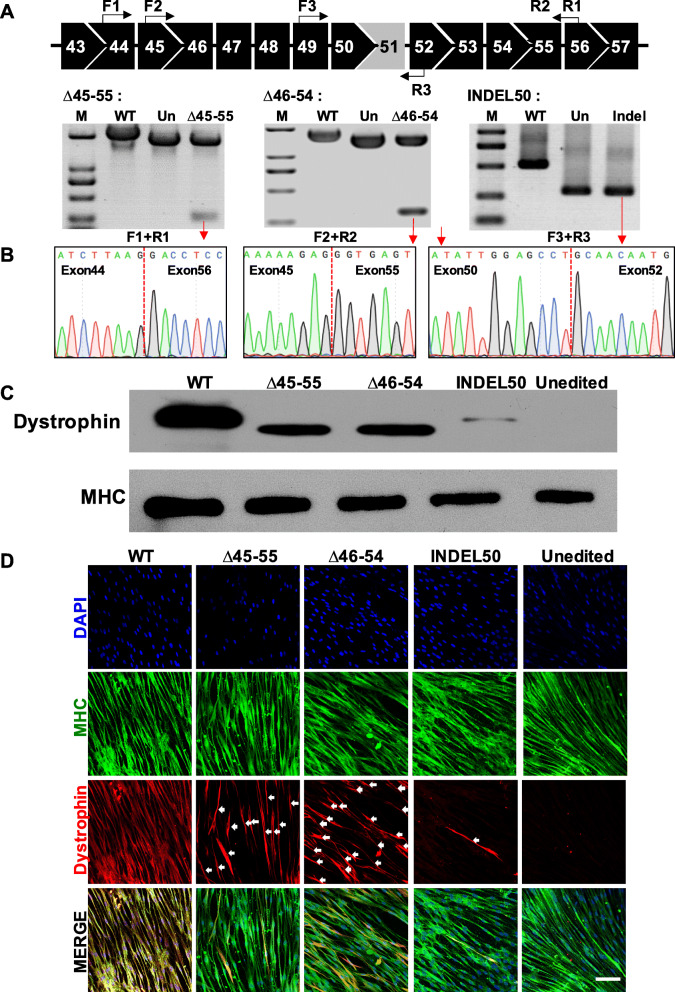
Restoration of dystrophin expression by CRISPR/Cas9. **a** CRISPR/Cas9-mediated gene editing restored dystrophin mRNA level in myotubes differentiated from DMD–MDSCs. Illustration of primer binding sites (black arrow) for RT-PCR (top row); all detected bands were of the expected size; red arrows indicate bands that were verified by Sanger sequencing in **b**. M, marker; Un, unedited. **b** The successful reframing of cDNA in the DMD–MDSCs subjected to three different gene-editing strategies was confirmed by Sanger sequencing. **c** Western blot analysis of dystrophin expression in targeted (∆45–55, ∆46–54, and INDEL50) or untargeted myotubes differentiated from the DMD–MDSCs; WT MDSCs served as a positive control. Expected molecular weights were 427 kDa for WT and INDEL50, 361 kDa for ∆45–55, and 375 kDa for ∆46–54; MHC served as a loading control. **d** Representative images of MHC (green) and dystrophin (red, white arrow) expression in targeted (∆45–55, ∆46–54, and INDEL50) and untargeted myotubes differentiated from the DMD–MDSCs as determined by immunocytochemistry; WT MDSCs served as a positive control, and nuclei were stained with DAPI (blue). Scale bar, 100 μm

To confirm CRISPR/Cas9-mediated restoration of dystrophin expression, we first examined the differentiation potential of the myotubes in the DMD–MDSCs. We differentiated the MDSCs into myotubes in vitro and examined the expression of the myogenic regulatory factors Myogenin, MyoD1, and Desmin, as well as the mature myotube marker myosin heavy chain (MHC), via reverse transcription (RT) PCR and immunofluorescence analysis (Additional file 1: Figure S6). The DMD–MDSCs targeted by the three distinct Cas9/gRNA gene-editing strategies were differentiated into myotubes, and the restoration of dystrophin protein expression was evaluated. Truncated mRNA transcripts were detected in the cells targeted with dual gRNAs (Fig. 3a). Sanger sequencing of the cDNA deletion band revealed the expected fusion of exons 44–56 and 45–55 with the ∆45–55 and ∆46–54 deletion strategies, respectively (Fig. 3b). For the INDEL50 strategy, in which a single gRNA was used to correct the aberrant reading frame by INDEL-mediated frameshifting, T-A cloning and sequencing of cDNA revealed that only 15% (3/20) of the transcripts were in frame (Additional file 1: Figure S7). Restoration of truncated dystrophin protein expression in the cells targeted with the three strategies was confirmed by Western blotting. As shown in Fig. 3c, the size of the truncated protein corresponding to the weight expected in the absence of exons 45–55 (∆45–55) and exons 46–54 (∆46–54) was approximately 361 and 375 kDa, respectively, whereas the molecular weight of full-length dystrophin was 427 kDa in both wild-type (WT) and INDEL50-targeted myotubes, although the latter showed only trace amounts of dystrophin. The intensity of ∆46–54 and ∆45–55 bands was higher than that of INDEL50 band. No dystrophin was detected in the proteins extracted from the DMD myotubes that had not been genetically edited (also see in Additional file 1: Figure S8).

The relative efficiency of the restoration of dystrophin protein expression in the myotubes by CRISPR/Cas9 was evaluated by immunocytochemistry. MHC was expressed in all myotubes. However, although dystrophin protein was detected in the myotubes differentiated from WT–MDSCs, it was absent in those differentiated from the DMD–MDSCs. When CRISPR/Cas9 worked, the expression of dystrophin was restored in the DMD–MDSC myotubes. We calculated the ratio of the number of dystrophin-positive fibers to that of MHC-positive fibers as a measure of in vitro therapeutic efficiency. As shown in Fig. 3d, ∆46–54/Cas9, ∆45–55/Cas9, and INDEL50/Cas9 restored the expression of dystrophin to varying degrees, whereas ∆46–54/Cas9 and ∆45–55/Cas9 were more efficient than INDEL50/Cas9 (also see in Additional file 1: Figure S9).

### CRISPR/Cas12a rescues dystrophin expression

*Streptococcus pyogenes* Cas9 (SpCas9), which is currently the most widely used Cas9 endonuclease, depends on a G-rich protospacer-adjacent motif PAM (NGG) that precludes genome editing of AT-rich regions. Previous studies have demonstrated that Cas12a, an RNA-guided endonuclease that prefers a T-rich PAM, is effective in mammalian genome editing [33, 39]. Thus, we used CRISPR/Cas12a to correct mutant *DMD* via deletion of exons 46–54 (Fig. 1c). Six Cas12a–gRNAs were designed to target introns 45 and 54 of *DMD*. The deletion efficiency of Cas12a in HEK293 cells combined with different gRNA pairs was evaluated with a dual-fluorescence reporter and by end-point PCR amplification of endogenous genomic DNA. Cas12a–gRNA pairs 1–4 exhibited the highest gene editing efficiency, and a low off-target frequency was selected for correction of the DMD–MDSCs (Additional file 1: Figures S10 and S11; Additional file 2: Tables S3 and S7).

**Fig. 4:**
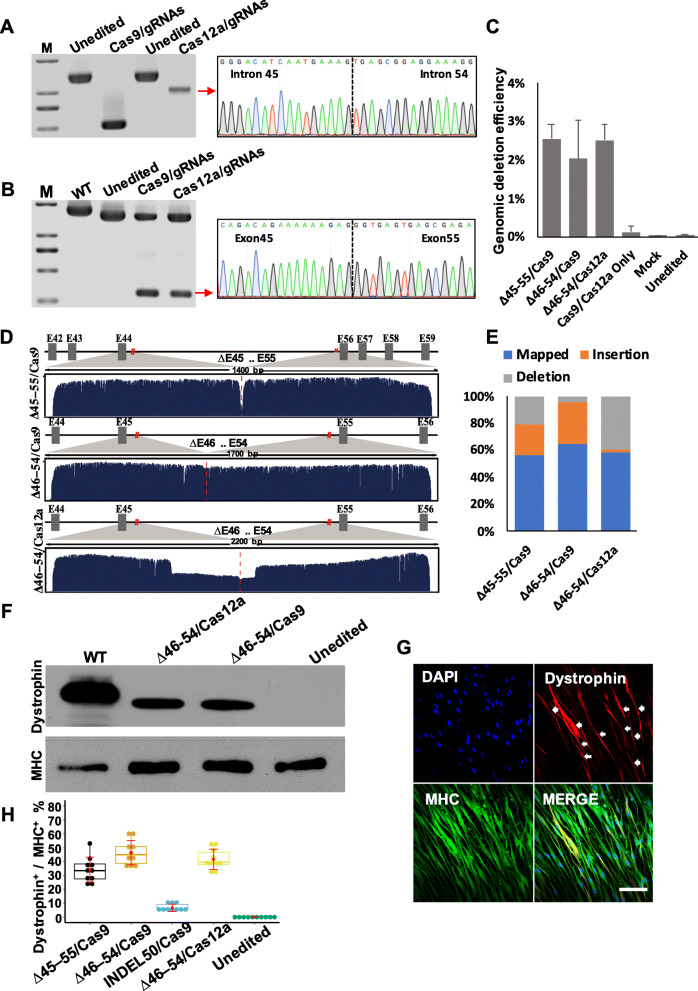
CRISPR/Cas12a -induced rescue of dystrophin expression. **a** PCR analysis of dystrophin expression in DMD–MDSCs targeted by Cas9/gRNA or Cas12a/gRNA specific to introns 45 and 54, respectively. All detected bands were of the expected size; the band indicated by a red arrow was verified by Sanger sequencing, which confirmed the junction of segmental introns 45 and 54. M, marker. **b** RT-PCR analysis of dystrophin expression in myotubes differentiated from the DMD–MDSCs targeted by Cas9/gRNA or Cas12a/gRNA, as shown in **a**. All detected bands were of the expected size; the band indicated by a red arrow was verified by Sanger sequencing, which confirmed the junction of exons 45 and 55. M, marker. **c** The editing efficiency of three large-scale excision strategies indicated by ddPCR assays. **d** Nanopore sequencing reads from edited MDSCs mapped at the spliced DMD genome; cut sites were marked by red lines. E, exon. **e** The base mutation percentage around the cut sites (40 bp for ∆45–55/Cas9, 65 bp for ∆46–54/Cas9 and ∆46–54/Cas12a). **f** Western blot analysis of dystrophin expression in targeted (∆46–54/Cas9 and ∆46–54/Cas12a) or untargeted myotubes differentiated from the DMD–MDSCs; WT MDSCs served as a positive control. Expected molecular weights were 427 kDa for WT and 375 kDa for Cas9/∆46–54 and Cas12a/∆46–54. MHC served as a loading control. **g** Representative images of MHC (green) and dystrophin (red, white arrow) expression in Cas12a/∆46–54-targeted myotubes differentiated from the DMD–MDSCs as determined by immunocytochemistry; nuclei were stained with DAPI (blue). Scale bar, 100 μm. **h** Representative box plots of the efficiency of the different gene-editing strategies in vitro as indicated by the ratio of the number of dystrophin-positive fibers (dystrophin^+^) to that of MHC-positive fibers (MHC^+^); ∆46–54/Cas9, ∆46–54/ Cas12a, and ∆45–55/Cas9 showed higher efficacy than INDEL50/Cas9 (*P* < 0.05, *n* = 10)

Expected deletion in genomic DNA and truncated mRNA transcripts were detected in ∆46–54/Cas9- and ∆46–54/Cas12a-targeted MDSCs, and Sanger sequencing confirmed the reframing of mutant *DMD* (Fig. 4a, b; Additional file 1: Figure S3C). ddPCR was performed to detect the large-scale excision efficiency mediated by Cas9 and Cas12a. Primer sets and probers were designed to target both the excised and the intact *DMD* gene (Additional file 2: Table S8). ddPCR indicated no significant difference among the three large-scale excision strategies meditated both by Cas9 and Cas12a, and over 2% of the *DMD* alleles had large-scale deletions (Fig. 4c; Additional file 1: Figure S12). Nanopore sequencing, a long-read sequencing technology, was performed to inspect the spliced *DMD* fragments and understand the splicing changes mediated by large-scale excision. About 2 kb regions nearby the cutting sites were amplified from the DMD–MDSCs edited by Cas9 or Cas12a with paired gRNAs. Over 50% of the fragments were observed to be exactly spliced on the cutting sites mediated both by Cas9 and Cas12a, whereas Cas12a was more prone to generating spliced fragments with deletions of about 500 bp (Fig. 4d, e; Additional file 1: Figures S13 and S14). The restoration of dystrophin protein expression was detected by Western blotting and immunocytochemistry (Fig. 4f, g; Additional file 1: Figure S15). We also evaluated the efficiency of the three Cas9- and Cas12a-mediated gene-editing strategies via double immunofluorescence labeling with antibodies against MHC and dystrophin and by calculating the ratio of the number of dystrophin-positive myotubes to that of MHC-positive myotubes. ∆46–54/Cas9 and ∆46–54/Cas12a both showed high efficiency (Fig. 4h), whereas *DMD* gene reframing by INDEL50/Cas9 was inefficient in restoring dystrophin. The efficiency of Cas12a-mediated ∆46–54 was comparable to that of Cas9-mediated ∆46–54.

### Off-target and transcriptome analyses

Although CRISPR-mediated gene editing is an effective approach for correcting disease-related gene mutations, mitigating the risk of off-target mutagenesis is critical for its application in gene therapy. Given that in silico prediction has poor sensitivity, high-throughput sequencing was conducted to assess the target specificity of both Cas9/gRNA and Cas12a/gRNA. The genomic DNA from the DMD–MDSCs targeted by Cas9/gRNAs and Cas12a/gRNA was analyzed via whole-exome sequencing. In contrast to the unedited DMD–MDSCs, multiple SNVs and indels were detected in both Cas9- and Cas12a-targeted cells with no significant difference (Additional file 1: Figure S16). Furthermore, 16 sites from ∆45–55/Cas9, 14 sites from ∆46–54/Cas12a, 8 sites from ∆46–54/Cas9, and 4 sites from INDEL50/Cas9 were selected for targeted NGS to validate the off-targets detected by whole-exome sequencing (Additional file 2: Table S9). The results showed a very low proportion of mutant reads in the targeted cells, and it was not significantly different from the controls (Additional file 1: Figures S17–S19). Given that the off-targeting effect of CRISPR is highly variable and hardly detected by whole-genome or whole-exome sequencing, GUIDE-seq [38] technology was conducted to capture all DNA double-stranded breaks (DSBs) generated by Cas9 and Cas12a. As we failed to capture any DSBs in the edited MDSCs, GUIDE-seq was performed using Cas9- or Cas12a-edited HEK293 cells as previously described. Dozens to hundreds of off-targets were detected within Cas9–gRNAs (Additional file 1: Figure S20). Although eight sites from IN54–Cas9–gRNA and one site from IN45–Cas9–gRNA were detected by GUIDE-seq as located in exons, high-throughput sequencing showed that those sites were not mutant in the MDSCs (Additional file 1: Figure S21). Taken together, whole-exome sequencing and GUIDE-seq indicated no detectable off-target activity in the MDSCs.

**Fig. 5:**
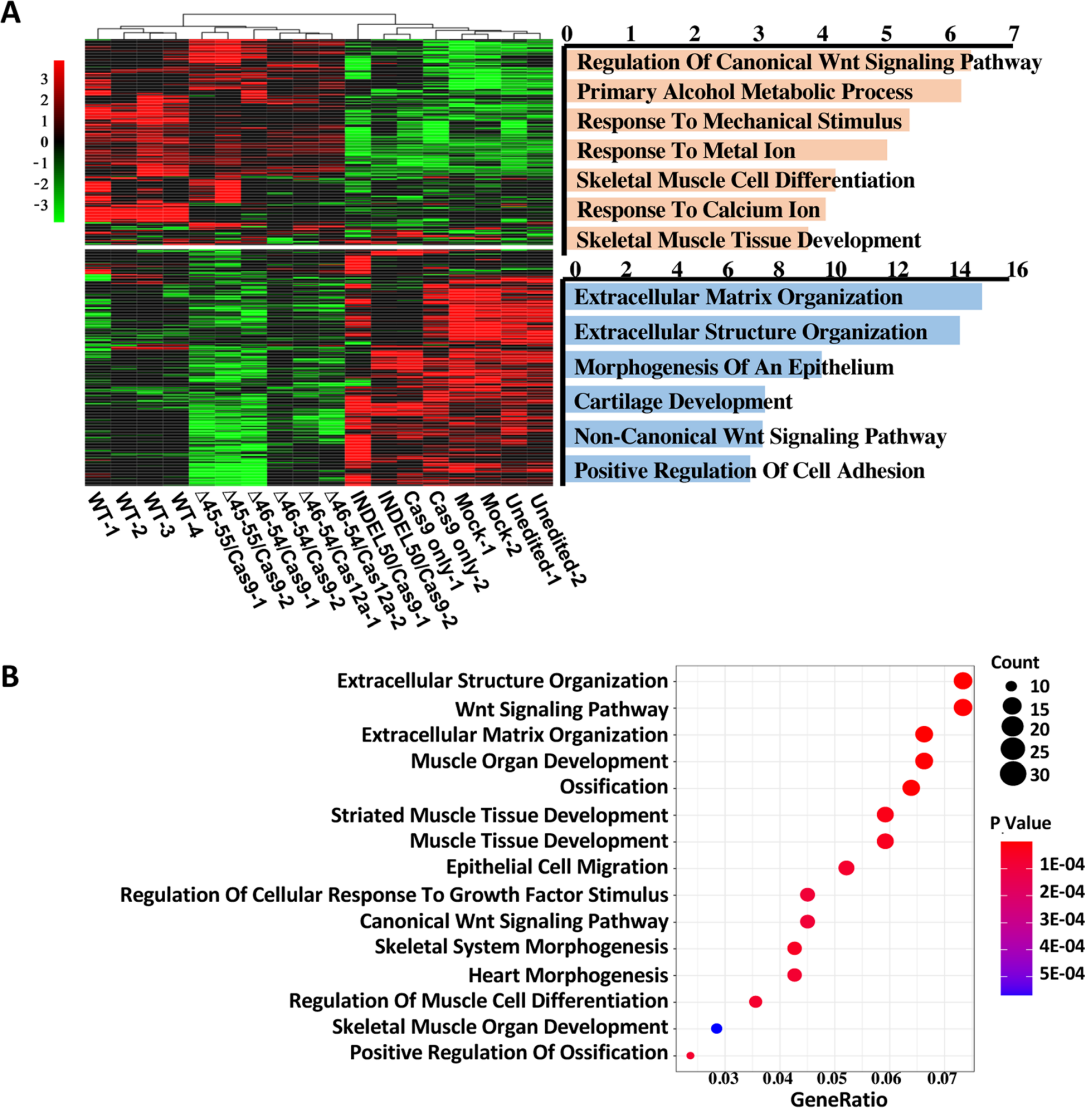
Transcriptome analyses. **a** Heatmap and hierarchical tree comparing the global transcriptome differences in edited (∆45–55/Cas9, ∆46–54/Cas9, ∆46–54/Cas12a and INDEL50/Cas9, *n* = 2) and negative control (Cas9 only, mock and unedited, *n* = 2) myotubes differentiated from the DMD–MDSCs with wild-type (WT, *n* = 4) myotubes differentiated from normal MDSCs. Red and green color intensities represent gene upregulation and downregulation, respectively. Gene ontology (GO) enrichment results of two clustered gene sets are shown on the right side of the heatmap; −log10 *p* values were used to produce the bar plots. **b** Gene Ontology analysis of transcripts in edited myotubes

To confirm the functional rescue of the myotubes derived from the edited DMD–MDSCs, we performed whole transcriptome analysis by RNA sequencing (RNA-seq). Hierarchical clustering (Fig. 5a) and principal component analysis (Additional file 1: Figure S22A) showed that the myotubes edited by large-scale-excision were significantly different from the negative controls but similar to the WT controls. Gene Ontology analysis revealed that transcripts that were highly enriched in the edited myotubes were related to muscle organ development, striated muscle tissue development, and muscle tissue development (Fig. 5b; Additional file 1: Figure S22B), suggesting that the rescued myotubes have a function more closely related to muscle function. In addition, differential expression analysis identified 715, 667, 973, and 51 differentially dysregulated genes in ∆45–55/Cas9, ∆46–54/Cas9, ∆46–54/Cas12a, and INDEL50/Cas9, respectively, compared with the negative controls (Additional file 1: Figure S23A). Together, these four strategies affected 28 genes. However, considering the limited therapeutic effect of INDEL50/Cas9, when it was excluded, the other three strategies affected a total of 469 genes (Additional file 1: Figure S23B), including the upregulated genes FOS and the downregulated genes COL6A1, IGF1, and TGFB1 (Additional file 1: Figures S23C and S23D). The expression of these genes in the edited myotubes was confirmed by Western blot (Additional file 1: Figure S23E). All of those genes are involved in muscle cell proliferation, differentiation, and muscle fibrosis and may affect muscle development and function.

### Generation of PDX DMD mouse model

**Fig. 6:**
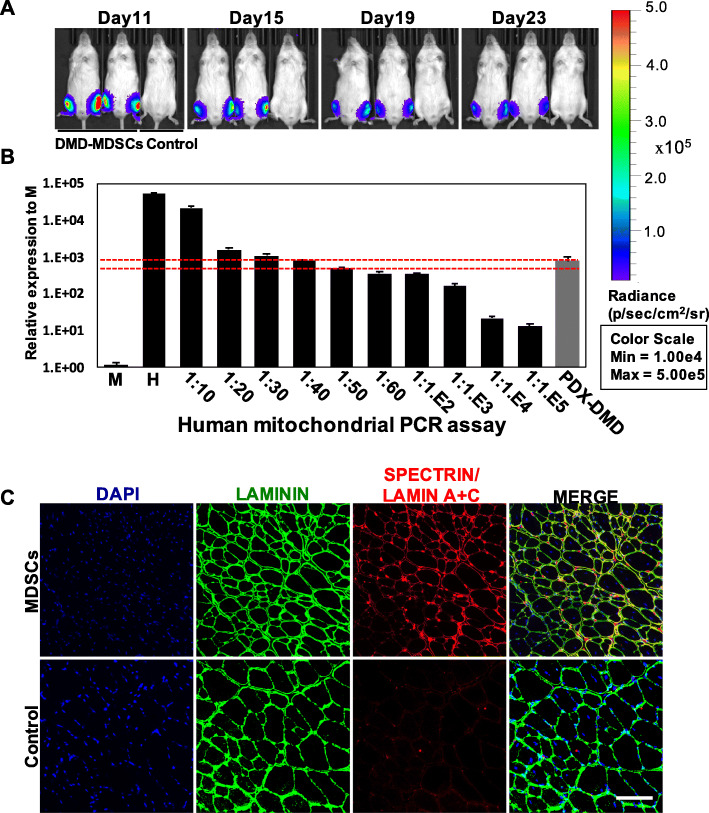
In vivo differentiation of DMD–MDSCs into muscle fibers. **a** DMD–MDSCs infected with lentivirus-expressing luciferase were transplanted into recipients, and engraftment was monitored by imaging over a period of 4 weeks. Bioluminescence images of representative injected mice were acquired on the indicated days. **b** qPCR analysis of human mitochondrial DNA revealed the presence of human cells in PDX DMD mouse following injection of DMD–MDSCs over a period of 4 weeks. Mouse DNA samples (M) and human DNA samples (H), as well as a series of mouse–human cell dilution samples, were run as negative and positive controls to estimate the degree of human cell contribution. The red dotted lines indicated that the proportion of human cells in the TA muscle of PDX DMD was between 2 and 2.5%. **c** Immunofluorescence staining of laminin (green), human spectrin (red signal in sarcolemma), and human Lamin A+C (red signal in nucleus) in the TA muscle of PDX DMD mice; nuclei were counterstained with DAPI (blue). Scale bar, 100 μm

Although CRISPR-mediated correction of the *DMD* gene has been demonstrated to be highly effective in cultured human cells, mouse models, and even a large animal model (i.e., dog), an important step for the clinical translation of this gene-editing strategy is to demonstrate its efficacy and safety in human muscle fibers in vivo. To this end, we transplanted the DMD–MDSCs into the TA muscle of NSI mice to generate a PDX DMD mouse model harboring human muscle fibers. We first examined changes in the transplanted MDSCs on the basis of luciferase activity. The MDSCs were infected with the lentivirus-expressing luciferase to conveniently monitor survival status in vivo. The NSI mice were treated with cardiotoxin to induce a niche for muscle fiber regeneration from human MDSCs (Additional file 1: Figure S24). On the second day, the MDSCs expressing luciferase were injected into TA muscles treated with cardiotoxin. Photon flow was evident from in vivo luminescence imaging at 4 weeks post transplantation, indicating that human MDSCs had settled in the NSI mice (Fig. 6a; Additional file 1: Figure S25; Additional file 2: Table S10). To further validate the presence of human cells, we performed a reported highly sensitive quantitative PCR assay based on the detection of human mitochondrial DNA, which allows for the detection of as few as one human cell in 10,000 mouse cells [37, 40]. The presence of human cells in the TA muscles injected with the DMD–MDSCs was approximately 2.5% (Fig. 6b). Moreover, human spectrin and Lamin A+C were detected in the muscle sections after 4 weeks (Fig. 6c), indicating that human MDSCs were able to differentiate into mature muscle fibers in mice. As shown in Fig. 6c, the human Lamin A+C-positive nuclei marking all human cells in the transplanted muscle always occurred with human spectrin-positive sarcolemma marking the differentiated human muscle fibers, showing high efficiency of myogenic differentiation of the DMD–MDSCs in vivo.

### In vivo gene editing of human DMD muscle fibers restores dystrophin expression and function

**Fig. 7:**
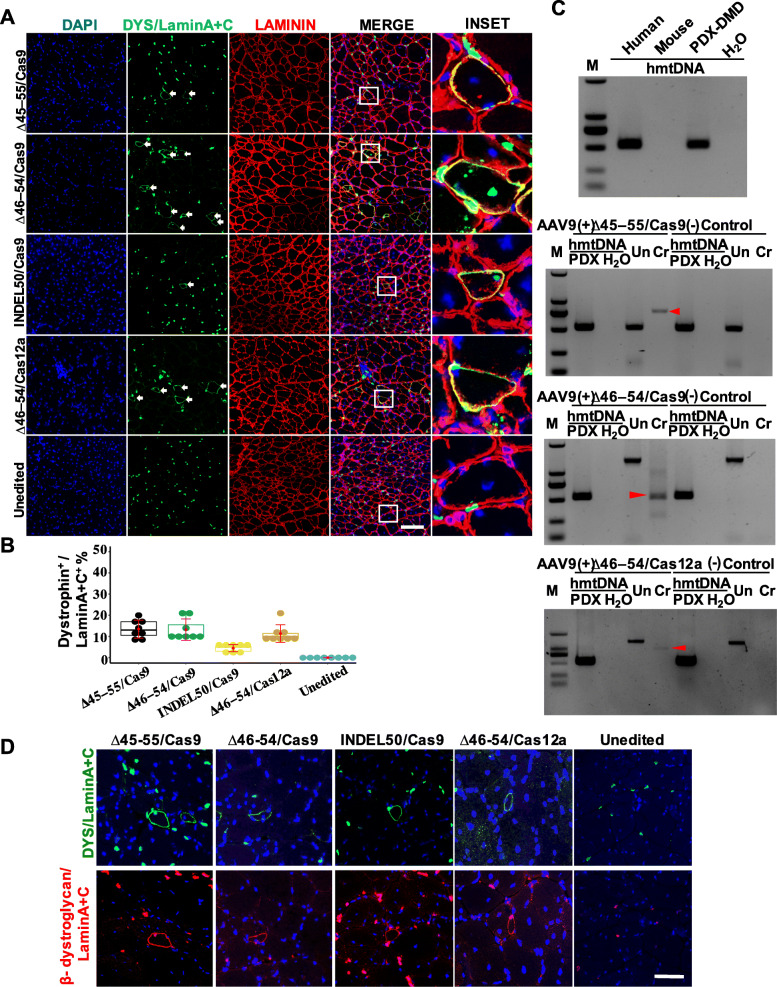
In vivo editing of human *DMD* gene restores dystrophin expression and localization in muscle fibers. (A) Immunofluorescence detection of human dystrophin (green signal in the sarcolemma, white arrow), human lamin A+C (green signal in the nucleus), and laminin (red) in the TA muscle of CRISPR-targeted PDX DMD mice; nuclei were counterstained with DAPI (blue). Mice without editing (unedited) served as the negative control. Scale bar, 100 μm. **b** Representative box plots of the therapeutic efficacy of the different gene-editing strategies in vivo as determined by the ratio of the number of dystrophin-positive fibers (dystrophin^+^) to that of lamin A+C-positive nuclei (LaminA+C^+^); ∆46–54/Cas9, ∆46–54/Cas12a, and ∆45–55/Cas9 showed higher efficacy than INDEL50/Cas9 (P < 0.05, *n* = 8). **c** Confirmation of the presence of human cells (upper left) and reframing of mutant DMD in PDX DMD mice by PCR. ∆45–55 yielded an intron 44/intron 55 junction (upper right), and ∆46–54 yielded an intron 45/intron 54 junction (bottom row). hmtDNA, human mitochondrial DNA. **d** β-Dystroglycan restoration in human muscle fibers treated with different gene-editing strategies. Human dystrophin and β-dystroglycan are visible as green and red signals, respectively; sections were stained with DAPI (blue) to identify nuclei and labeled with an antibody against human Lamin A+C (green or red) to identify human nuclei. Mice without editing (unedited) served as a negative control. Scale bar, 50 μm

We next examined the capacity of CRISPR/Cas9- and CRISPR/Cas12a-mediated gene editing to correct the mutant *DMD* gene in vivo. We first evaluated in vivo gene transfer to muscle fibers mediated by AAV9 vector. At 1 week post infection, EGFP expression was detected in the injected muscle tissue, indicating the robust delivery of AAV9 (Additional file 1: Figure S26). To evaluate the possibility of correcting DMD muscle fibers in vivo, we packaged Cas9/gRNA and Cas12a/gRNA targeting various loci in the human *DMD* gene into AAV9 vectors and delivered to the TA muscle via intramuscular injection 2 weeks after transplantation of the DMD–MDSCs into the NSI mice. After 4 weeks, human dystrophin expression was detected in the muscle tissues (Fig. 7a; Additional file 1: Figure S27). Considering the robust myogenic differentiation of the DMD–MDSCs in vivo, we calculated the ratio of the number of human dystrophin-positive fibers to that of human Lamin A+C-positive nuclei as a measure of in vivo relative therapeutic efficiency. ∆45–55/Cas9, ∆46–54/Cas9, and ∆46–54/Cas12a were highly efficient in rescuing dystrophin expression in vivo (Fig. 7b), consistent with the in vitro results. We also detected the expected bands in ∆45–55- and ∆46–54-targeted mice via PCR of the genomic DNA extracted from the treated muscles. The results confirmed the genome editing of human muscle fibers in the PDX DMD mice (Fig. 7c). In addition, we examined the expression of the DGC component β-dystroglycan to determine the functionality of the rescued myotubes. Dystrophin and β-dystroglycan were correctly localized in all rescued human muscle fibers (Fig. 7d), demonstrating that DGC function was restored and the DMD muscle fiber membrane was stable.

## Discussion

In the present study, we used CRISPR-mediated gene-editing technology to repair functional human muscle fibers in a mouse model of DMD by using patient-derived primary MDSCs. A comparison of various gene-editing strategies mediated by particular gRNAs was performed in the DMD–MDSCs and the PDX model, and highly efficient strategies were identified.

CRISPR, which is adapted from bacterial acquired immune surveillance system, is a useful tool for genome editing and the treatment of various inherited diseases. Previous studies have shown that it can be used to modify the *DMD* gene in the skeletal myoblasts of DMD patients [20, 41], induced pluripotent stem cells (iPSCs) [21, 22], and in the germ line of DMD mice [42]. The CRISPR/Cas9 system can also enable the rescue of dystrophin protein expression in mdx [23–25], mdx^4cv^ [43] and genetically humanized mice [28], and in dogs [27] by using AAV vectors or in vivo electroporation. In all of these cases, CRISPR-mediated gene editing permanently restored dystrophin protein expression to alleviate the clinical symptoms of DMD, yielding a phenotype more closely resembling that of BMD.

Previous studies did not address whether mutant dystrophin in DMD patient-derived muscle cells could be edited in vivo via gene editing. We addressed this question by using a PDX model, which more authentically mimics human pathophysiological processes, response to therapy, and provides a sensitive indicator of in vivo efficacy. PDX mouse models have been established by transplanting seed cells, such as lymphocytes [44], hematopoietic stem cells [44], hepatic cells [45], and cancer cells [46], into immunodeficient mouse. We used human primary MDSCs, which are characterized by high proliferative and myogenic differentiation capacities and low immunogenicity [34], from a DMD patient as the seed cells for heteroplastic transplantation into immunodeficient NSI mice that lack B/T lymphocytes and natural killer cells [47, 48]. The transplanted DMD–MDSCs differentiated into mature muscle fibers in vivo, indicating successful establishment of the PDX DMD mouse model. CRISPR/Cas9 was previously used to target genomic mutations in mouse PDX tumor models [49–51] in which cancer cells exhibited unlimited proliferation, which is very different from terminally differentiated muscle cells. The present study is the first to apply CRISPR-mediated genome editing of a specific genomic locus (the *DMD* gene) in adult human nontumor cells (i.e., MDSCs) for the restoration of protein expression and function in vivo.

Cas9 is currently the most widely used nuclease for gene editing. However, its application is limited by the fact that Cas9-mediated cleavage requires a G-rich PAM at the 3′ end of the protospacer for its editing function, whereas introns harbor T-rich regions. In contrast to Cas9, Cas12a recognizes T-rich PAM sequences at the 5′ end of target DNA sequences, which allows targeting of regulatory regions and introns in AT-rich genomes. This useful property expands the range of disease-related mutations that can be edited with the CRISPR system [52]. Moreover, Cas12a is a highly specific programmable nuclease that can reduce off-target effects compared with Cas9 [39]. Our results revealed that ∆46–54/Cas12a-gRNAs have fewer off-target sites than ∆46–54/Cas9–gRNAs, and the deletion efficiency of Cas12a is comparable to that of Cas9. Thus, Cas12a provides an alternative nuclease for future gene therapy in DMD patients.

The efficiency of gene-editing strategies employed in previous studies is unclear due to the use of immortalized myoblasts or iPSCs derived from DMD patients. Herein, we designed a novel method for reframing mutant *DMD* by deletion of exons 46–54, which encompasses nearly 60% of DMD mutations, and compared the efficiency of this and previously reported approaches both in vitro and in vivo. ddPCR and high-throughput sequencing of the PCR products of the edited *DMD* gene indicated that the four strategies we compared follow a same trend of reframing efficiency. However, RNA-seq and immunofluorescence showed significantly low efficiency of INDEL-50 mediated restoration of dystrophin. High-throughput sequencing results from the E50-gRNA target indicated that about 30% of the reframed reads were unable to transcribe the correct dystrophin, which reduced the dystrophin restoration efficiency of the INDEL50 strategy. Thus, highly efficient gRNA for introducing indel-mediated DMD must be designed. gRNAs targeting the intron–exon junction area can substantially affect 5′ or 3′ splice sites and reduce the recovery efficiency of INDEL strategy. These results indicated that the large-fragment deletion strategies we used were efficient in vivo and might meet the requirements of clinical efficacy. However, considering that the targeting efficiency of gRNAs is crucial to restoring dystrophin expression, the comparisons are only valid for these particular gRNAs and cannot be extended to these approaches in general.

## Conclusions

In summary, this study provides evidence for the efficacy of in vivo genome editing to correct disruptive mutations and restore dystrophin expression and function in DMD patient-derived muscle fibers. The PDX DMD mouse model can be used to screen potentially effective strategies for clinical application. The continued studies of gene-editing strategies, gene delivery approach, toxicology, and immunology in large animals will provide further insights into the potential application of gene therapy for DMD.

## Supplementary Information

**Additional file 1: Supplementary Figures S1-S27.**

**Additional file 2: Supplementary Tables S1-S10.**

## Data Availability

The sequencing data reported in this paper is deposited in GEO, accession number GSE168007 (https://www.ncbi.nlm.nih.gov/geo/query/acc.cgi?acc=GSE168007) [53].
