# Supplementary material for: In vivo genome editing in mouse restores dystrophin expression in Duchenne muscular dystrophy patient muscle fibers
Source: Genome Med. 2021 Apr 12;13:57. doi: 10.1186/s13073-021-00876-0 (PMC8042958; doi:10.1186/s13073-021-00876-0)
Supplement: Supplementary file 1 — Additional file 1: Supplementary Figures S1-S27. [file 13073_2021_876_MOESM1_ESM.pdf]

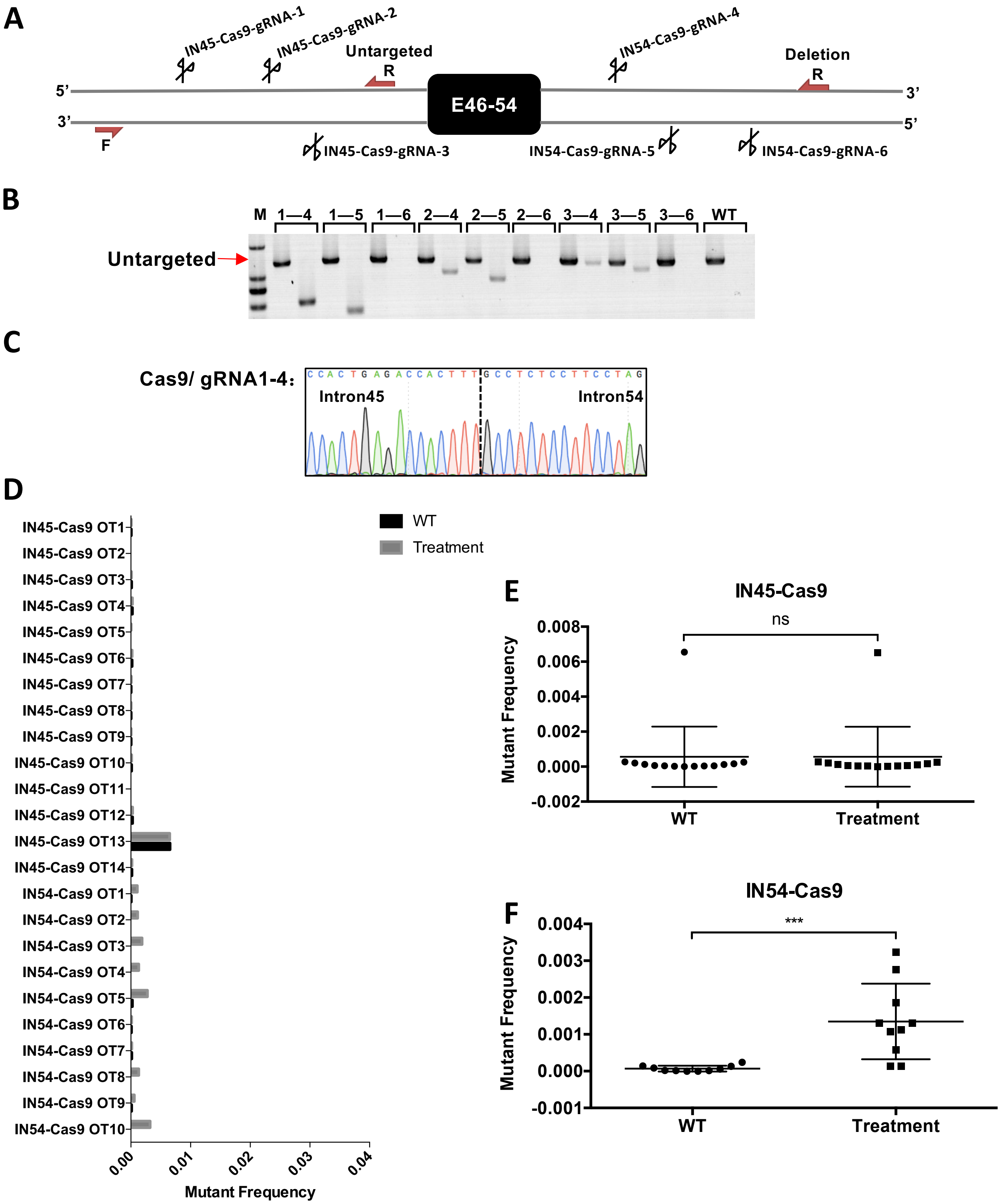

Figure S1. End-point PCR performed using genomic DNA from Cas9/gRNA-targeted HEK293 cells and off-target analysis by deep sequencing. (A) Schematic illustration of the gRNAs targeting introns 45 and 54 of the *DMD* gene. Scissors indicated gRNA target sites, and the red arrow indicates the primer-binding sites for the end-point genomic PCR in (B). (B) End-point genomic PCR using DNA extracted from HEK293 cells transfected with Cas9/gRNAs; bands under the Untargeted bands (red arrow) in the column for each gRNA combination correspond to the removal of exons 46 to 54. #(1–3)–#(4–6), gRNA combinations; M, marker; WT, wild type. (C) PCR products from (B, 1–4) were cloned and sequenced to confirm the junction of introns 45 and 54. (D, E, F) Off-target analysis of Cas9/gRNA1–4-targeted HEK293 cells by deep sequencing. OT, off target; WT, wild type. Paired two-tailed t-test was applied (***P < 0.001,ns, not significant) .

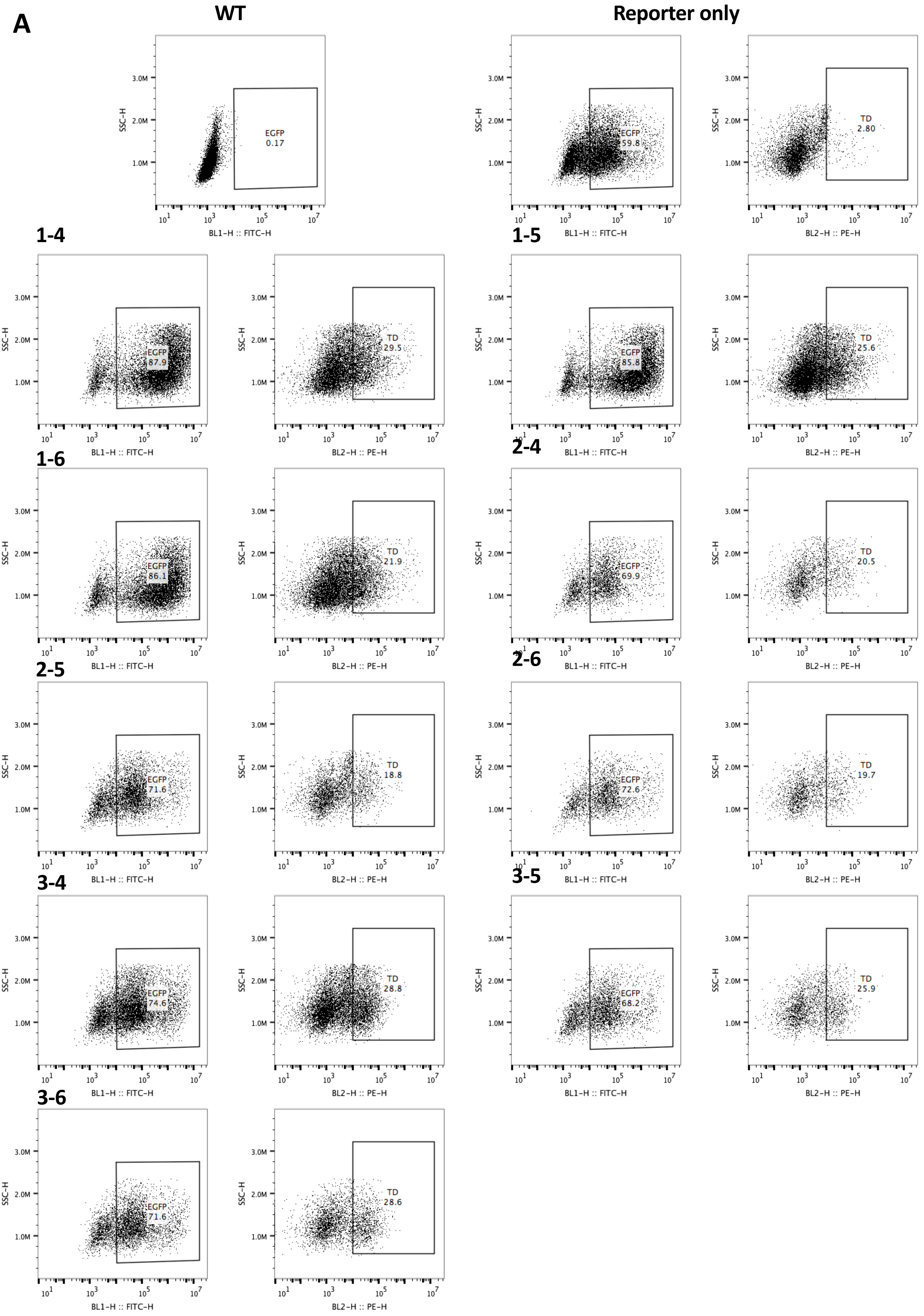
A
WT
Reporter only
EGFP 0.17
EGFP 59.8
TD 2.80
1-4
EGFP 87.9
TD 29.5
1-5
EGFP 85.8
TD 25.6
1-6
EGFP 86.1
TD 21.9
2-4
EGFP 69.9
TD 20.5
2-5
EGFP 71.6
TD 18.8
2-6
EGFP 72.6
TD 19.7
3-4
EGFP 74.6
TD 28.8
3-5
EGFP 68.2
TD 25.9
3-6
EGFP 71.6
TD 28.6
SSC-H
3.0M
2.0M
1.0M
BL1-H :: FITC-H
BL2-H :: PE-H

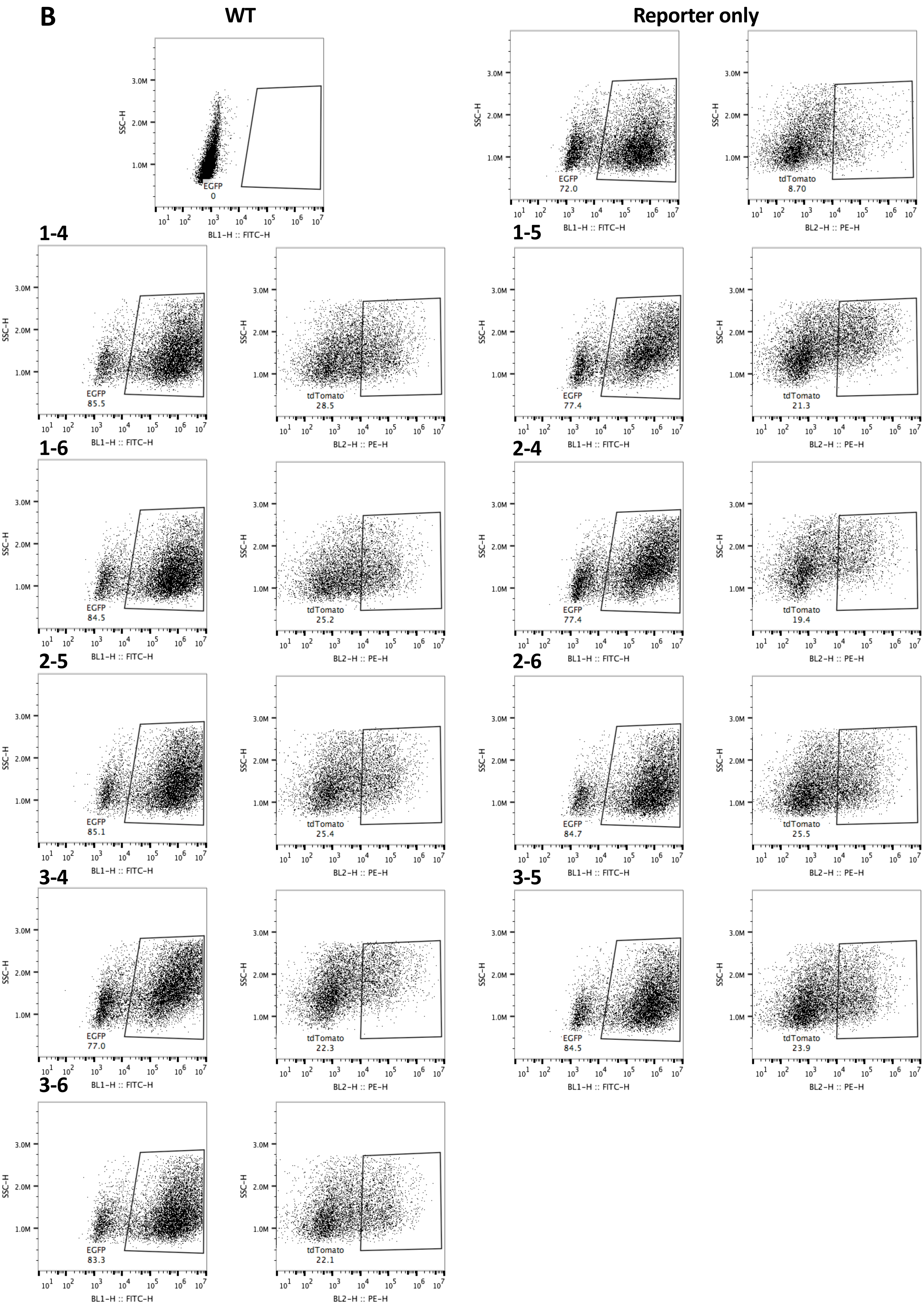

Figure S2. Flow cytometry of HEK293 cells co-transfected with the reporter construct and Cas9/gRNA.（A,B）2 biological replicates.

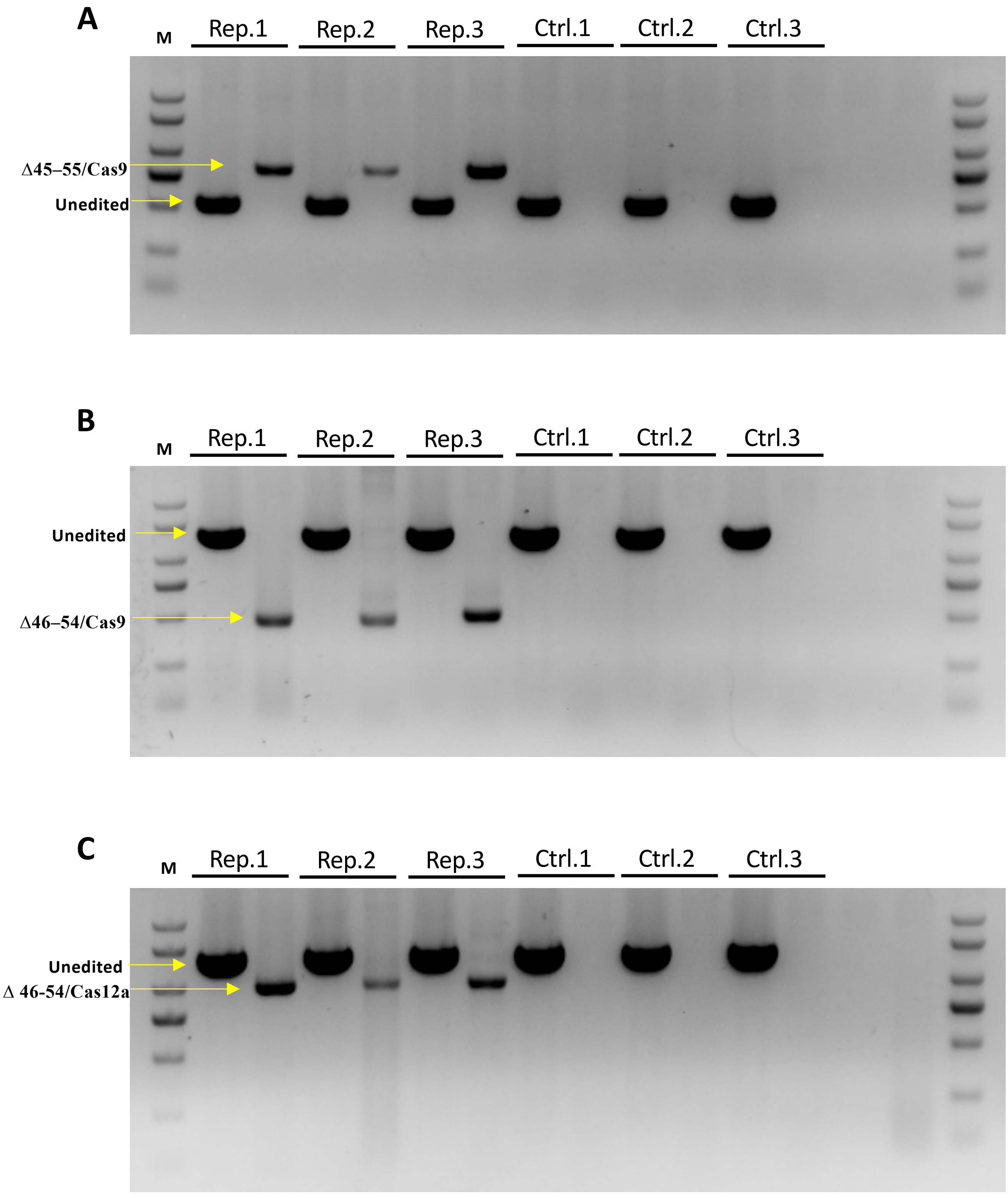

Figure S3. Replicates of PCR and electrophoresis for reframed *DMD* of (A) Δ45–55/Cas9, (B) Δ46–54/Cas9 and (C) Δ46-54/Cas12a edited MDSCs. M, marker; Rep, replicate; Ctrl, control.

A

Exon 50	gRNA	PAM	Intron 50		

```
5′-CTAGCTCCTGGACTGACCACTATTGGAGCCTgtaag-3′
3′-GATCGAGGACCTGACTGGTGATAACCTCGGAcattc-5′
```

Sequence	Clones	Frame
CTAGCTCCTGGACTGACCACTATTGGAGCCTgtaag	(x14)	
CTAGCTCCTGGACTGAC-ACTATTGGAGCCTgtaag		+2
CTAGCTCCTGGACTGACCACTATTGGAGT-CTgtaag	(x2)	+3
CTAGCTCCTGGACTGACCACTG-TTGGAGCCTgtaag		+3
CTAGCTCCG-GGACTGACCACTATTGGAGCCTgtaag		+3
CTAGCTCCTGGACTGACC--TATTGGAGCCTgtaag		+1
CTAGCTCCTGGACTGACCA-TATTGGAGCCTgtaag		+2
CTAGCTCCTGGACTGACCTAGCTCC-----TGGAGCCTgtaag		+2
CTAGCTCCTGGACTGACC------------Tgtaag		+3
CTAGCTCCTGGACTGACCACTTATTGGAGCCTgtaag		+1
CTAGCTCCTGGACTG----------GAGCCTgtaag		+2
CTAGCTCCTGGACTGACCACTGTATTGGAGCCTgtaag		+2
CTAGCTCCTGGACTGACCACCTATTGGAGCCTgtaag		+1
CTAGCTCCT---------------GGAGCCTgtaag		+3
CTAGCTCCTGGACTGACC-----------CTgtaag		+1
CTAGCTCCTGGACTGACCA------------gtaag		+3

B

Total edit events: 16/30 (53.3%)

+1 Frame:4/30 (13.3%)

+2 Frame: 5/30 (16.7%)

+3 Frame: 7/30 (23.3%)

Figure S4. Targeted frameshifts to restore the reading frame of dystrophin by CRISPR/Cas9. (A) The 3' region of exon 50 was targeted by gRNA (blue); the PAM (green) is located at the 3' end of the protospacer. The exon 50 locus was PCR-amplified from DMD-MDSCs treated with Cas9/gRNA targeting exon 50. Sequences of individual clones were confirmed by Sanger sequencing. The sequence in the third row is the unmodified sequence; the number of clones of each sequence is indicated in parentheses, and changes in the sequence are indicated in red. (B) Summary of gene editing efficiency and total reading frame conversions resulting from gene modifications shown in (A).

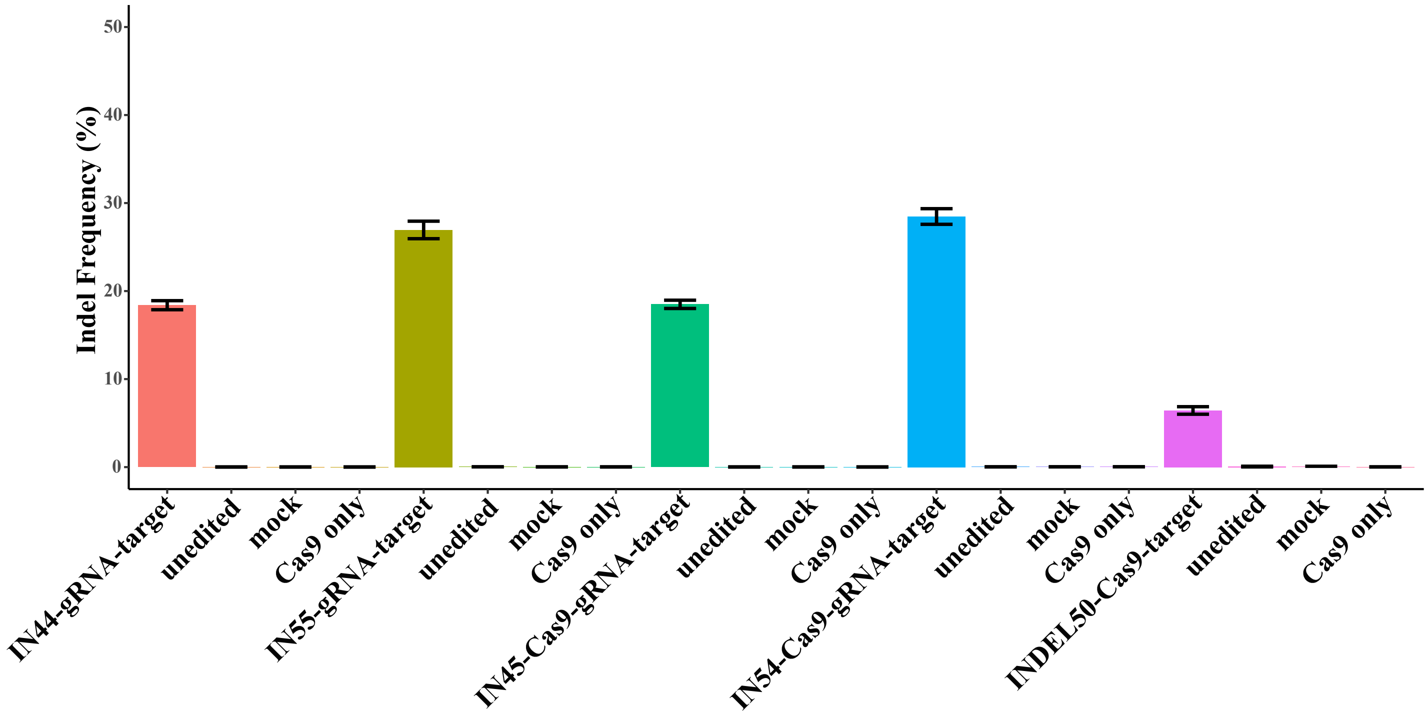

Figure S5. Targeted NGS detect the ratio of indels generated by gRNAs.

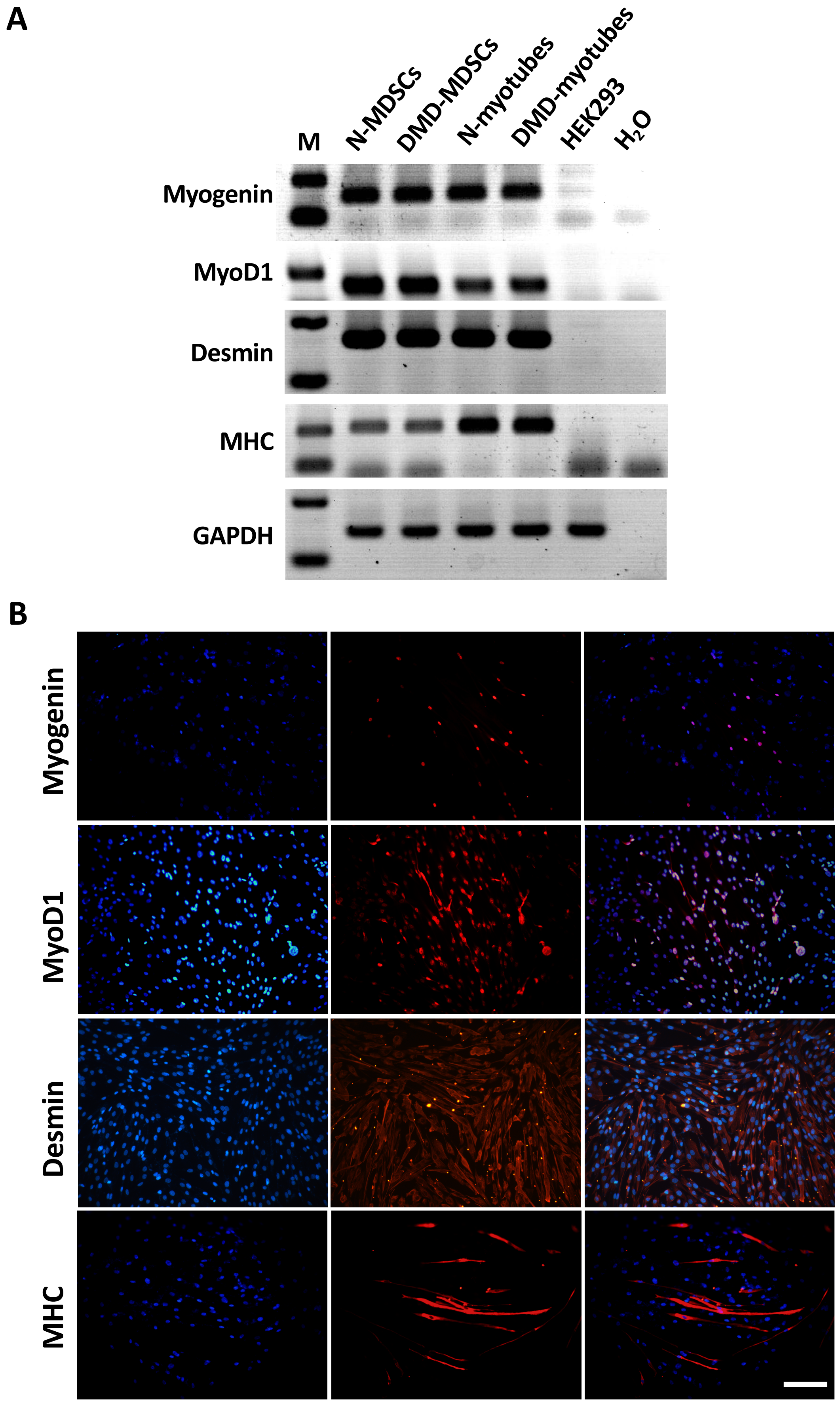

Figure S6. Identification of human MDSCs. (A) RT-PCR analysis of Myogenin, MyoD1, Desmin, and MHC expression in normal or DMD-MDSCs and differentiated myotubes. (B) Representative images of DMD-MDSCs and differentiated myotubes expressing Myogenin, MyoD1, Desmin, and MHC, as detected by immunocytochemistry. Scale bar, 100 µm.

A

```
        Exon 50                               PAM        Exon 52
5′-CAGCCTGACCTAGCTCCTGGACTGACCACTATTGGAGCCT|GCAACAATGC-3′
3′-GTCGGACTGGATCGAGGACCTGACTGGTGATAACCTCGGA|CGTTGTTACG-5′

   CAGCCTGACCTAGCTCCTGGACTGACCACTATTGGAGCCT|GCAACAATGC     (x12)   Frame
   CAGCCTGACCTAGCTCCTG-ACTGACCACTATTGGAGCCT|GCAACAATGC              +2
   CAGCCTGACCTAGCTCCTGGACTGACCA-TATTGGAGCCT|GCAACAATGC              +2
   CAGCCTGACCTAGCTCCTGGACTGACCACTTTATTGGAGCCT|GCAACAATGC  (x2)      +2
   CAGCCA-GACCTAGCTCCTGGACTGACCACTATTGGAGCCT|GCAACAATGC             +3
   CAGT-CTGACCTAGCTCCTGGACTGACCACTATTGGAGCCT|GCAACAATGC             +3
   CAGCCC-GACCTAGCTCCTGGACTGACCACTATTGGAGCCT|GCAACAATGC             +3
   CAGCCTGACCTAGCTCCTGGACTGACCACTATTGGG-GCCT|GCAACAATGC             +3
```

B

Total edit events: 8/20 (40%)

+1 Frame: 0/20 (0%)
+2 Frame: 1/20 (5%) (with stop codons)
+2 Frame: 3/20 (15%) (without stop codons)
+3 Frame: 4/20 (20%)

Figure S7. Targeted frameshifts to restore the cDNA reading frame of *DMD* by CRISPR/Cas9-mediated gene editing. (A) The 3' region of exon 50 was targeted by gRNA (blue); the PAM (green) is located at the 3' end of the protospacer. The exon 50 locus in myotubes differentiated from DMD-MDSCs transfected with Cas9/gRNA targeting exon 50 was amplified by RT-PCR. Sequences of individual clones were confirmed by Sanger sequencing. The sequence in the third row is the unmodified sequence; the number of clones of each sequence is indicated in parentheses, and changes in the sequence are shown in red. (B) Summary of gene editing efficiency and total reading frame conversions resulting from gene modifications shown in (A).

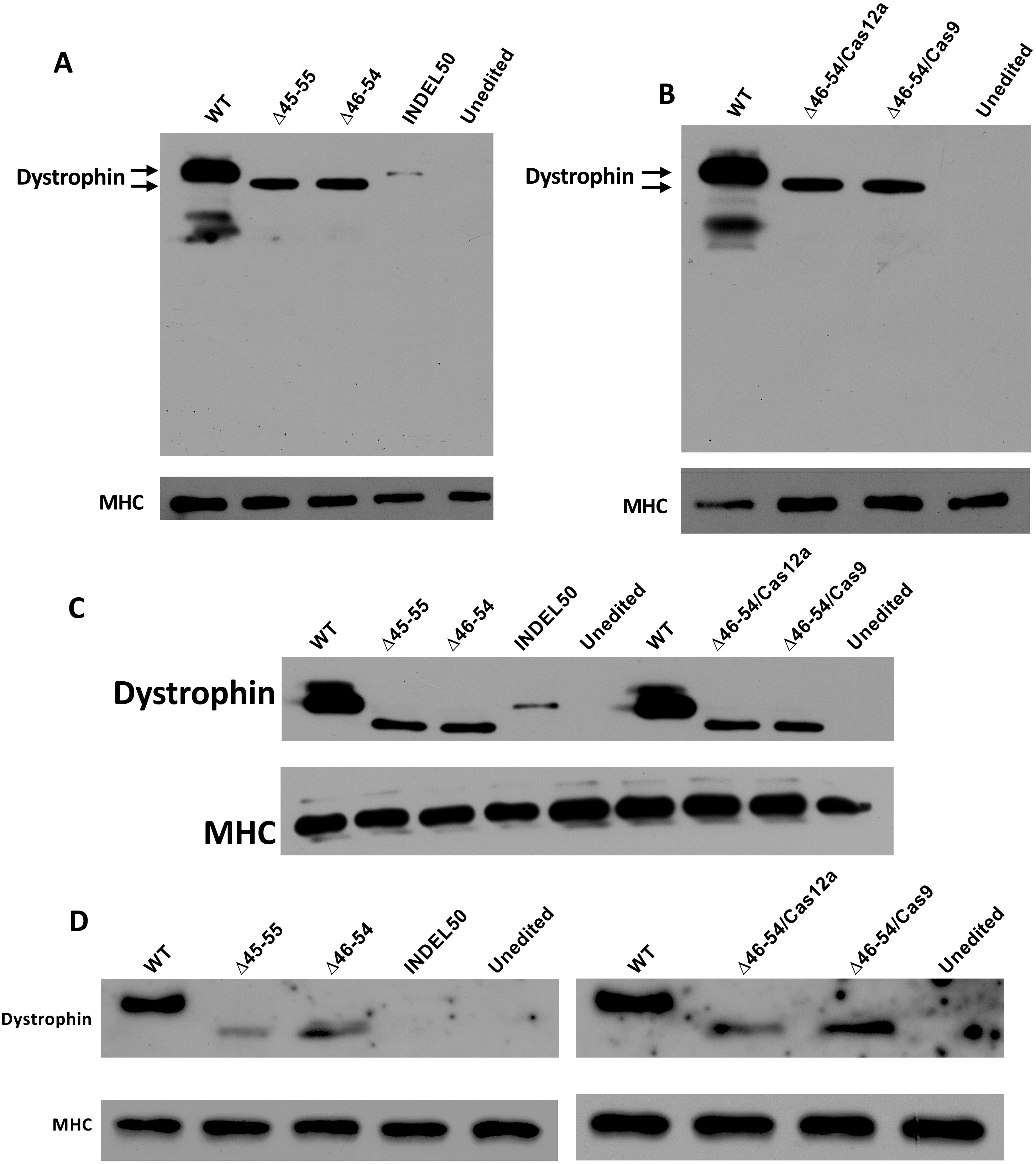

Figure S8. Western blot images of dystrophin expression. (A) reference to Fig3C , (B) reference to Fig4F; (C,D) replications of the Western blot images of dystrophin expression.

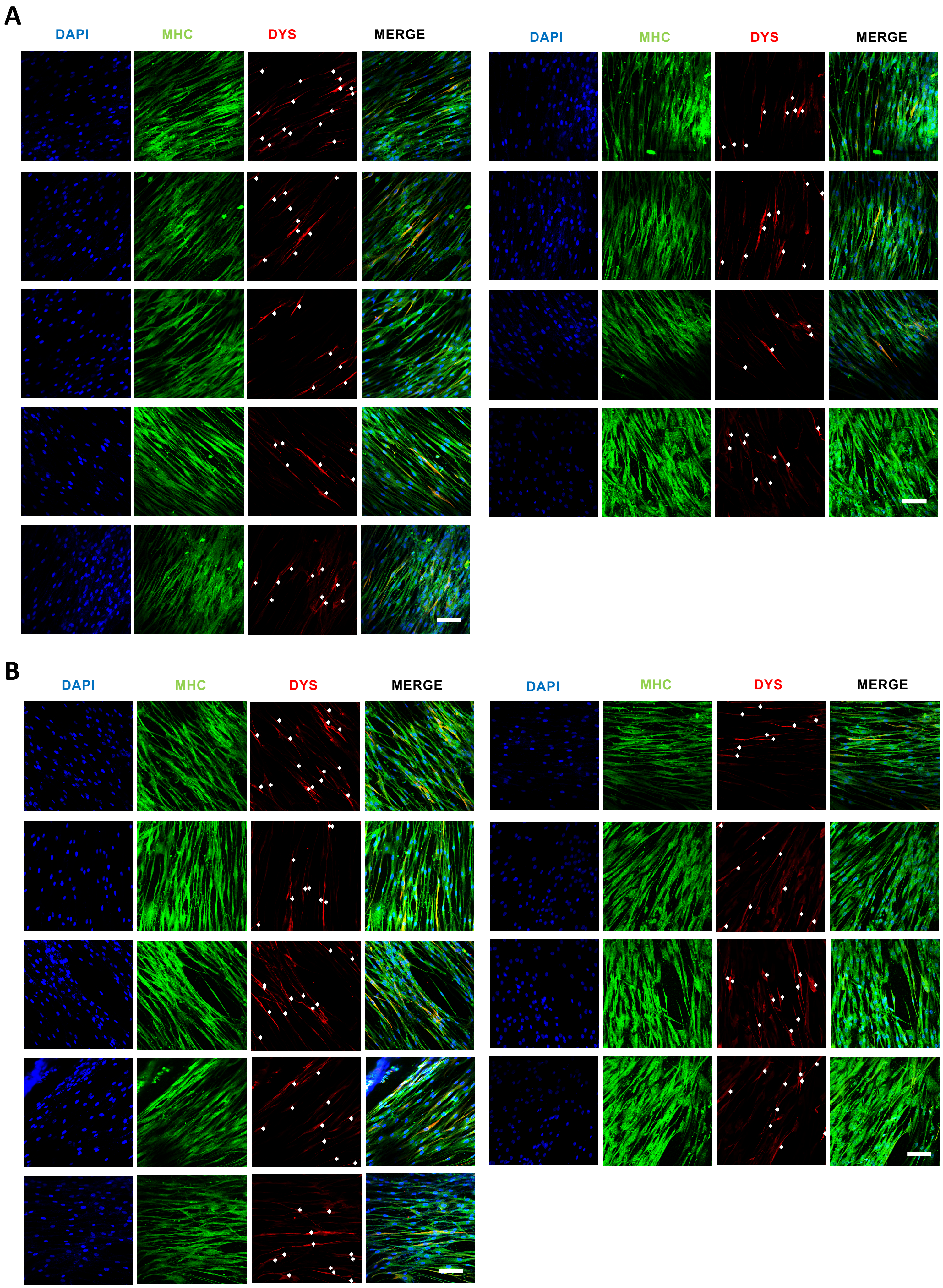
A
DAPI
MHC
DYS
MERGE
DAPI
MHC
DYS
MERGE
B
DAPI
MHC
DYS
MERGE
DAPI
MHC
DYS
MERGE

C

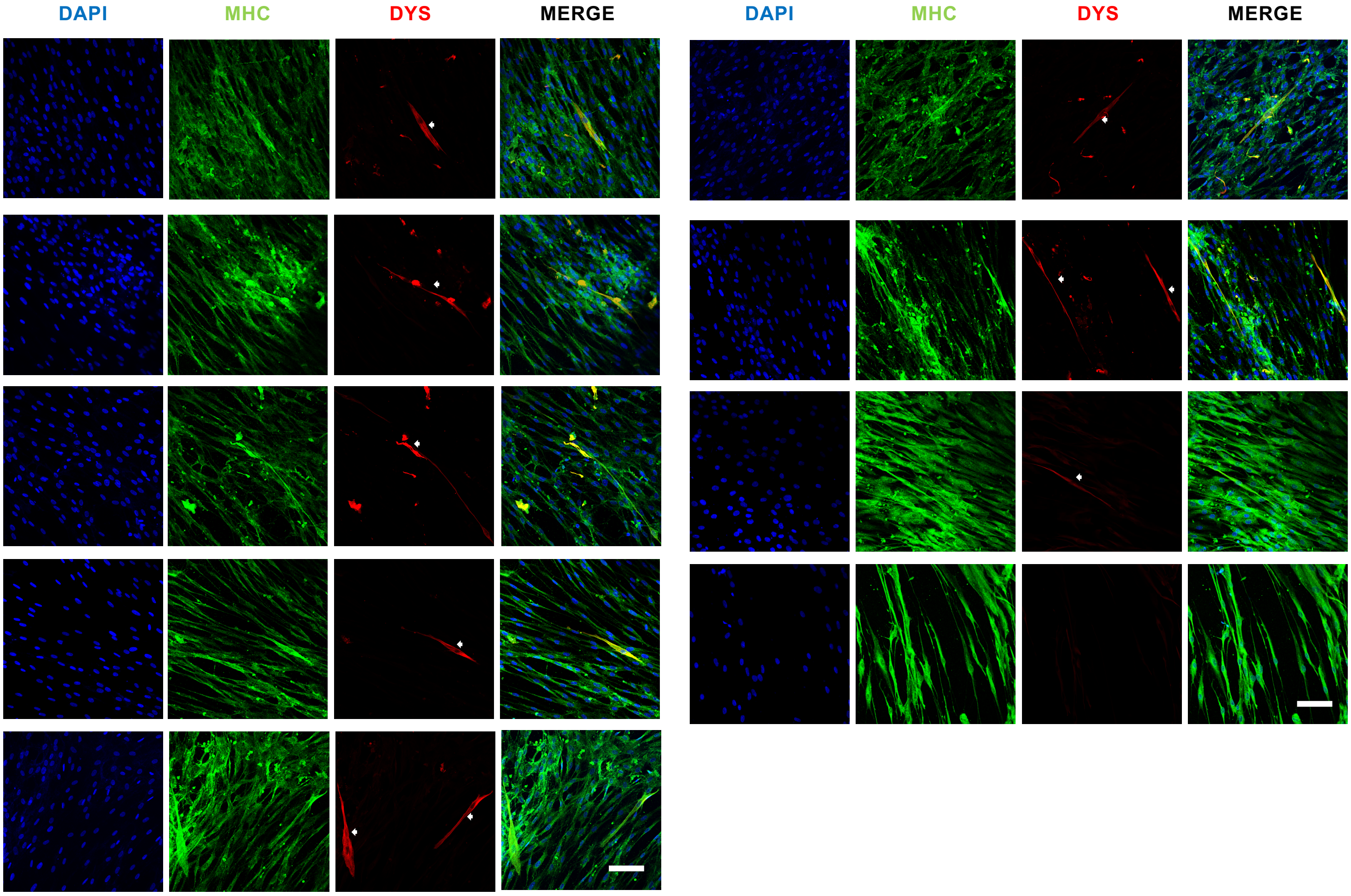

Figure S9. Supplementary images of MHC (green) and dystrophin (red, white arrow) expression in (A) Δ45–55, (B) Δ46–54 and (C) INDEL50-edited myotubes differentiated from DMD-MDSCs, as determined by immunocytochemistry; nuclei were stained with DAPI (blue). Scale bar, 100 μm.

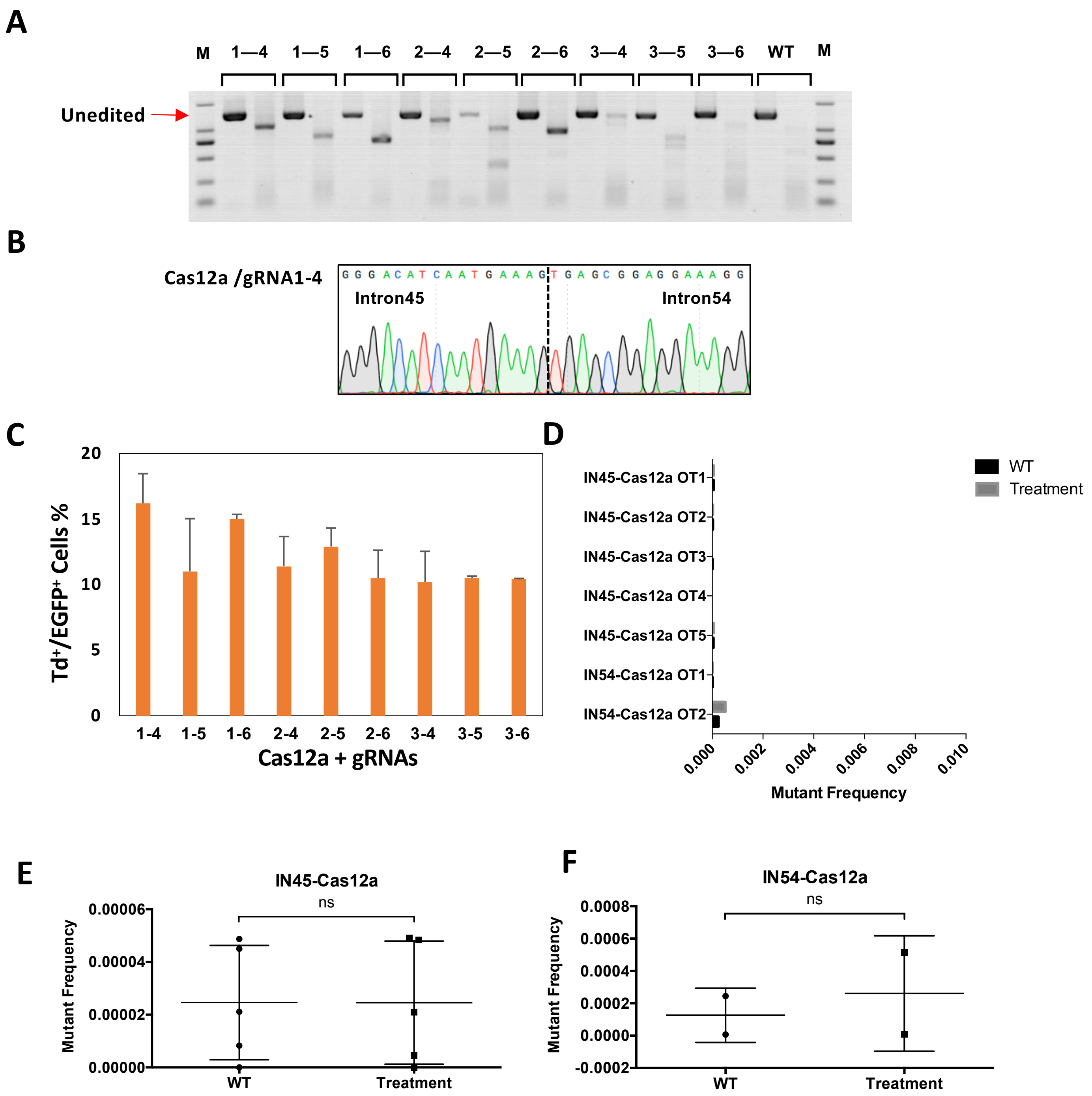

Figure S10. Screen of gRNAs for CRISPR/Cas12a targeting of the *DMD* gene and off-target analysis by deep sequencing. (A) End-point genomic PCR analysis of DNA extracted from HEK293 cells transfected with Cas12a/gRNAs; bands labeled as Untargeted (red arrow) for each gRNA combination indicate the removal of exons 46–54. M, marker; #(1–3) and #(4–6), gRNA combinations. (B) PCR products 1–4 from (A) were cloned and sequenced to confirm the joining of introns 45 and 54. (C) Flow cytometry analysis of HEK293 cells co-transfected with the reporter construct and Cas12a/gRNA (n = 2). (D, E, F) Deep sequencing off-target analysis of HEK293 cells transfected with Cas12a/gRNA1–4. OT, off target. Paired two-tailed t-test was applied (ns, not significant) .

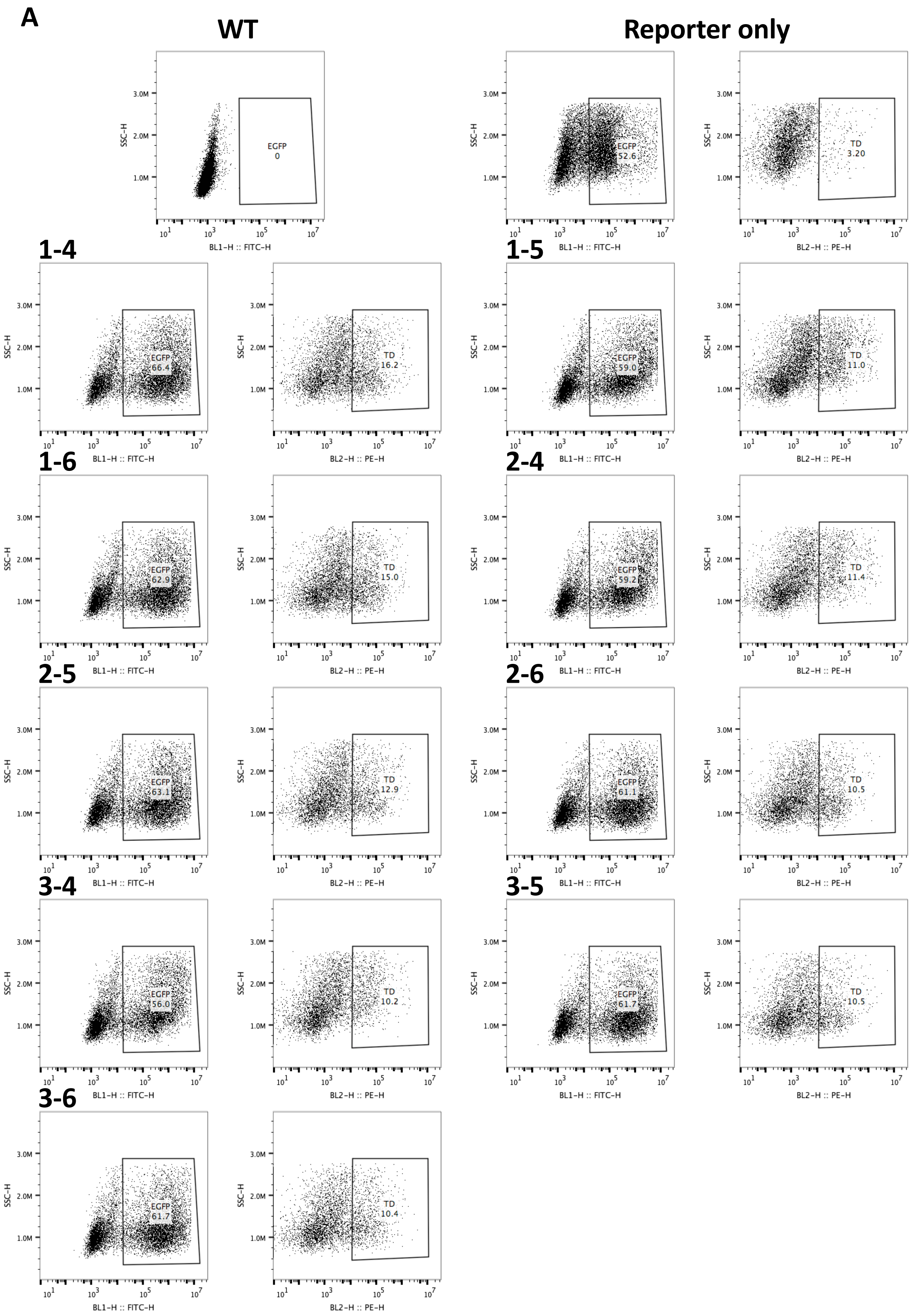
A
WT
Reporter only
EGFP 0
EGFP 52.6
TD 3.20
1-4
EGFP 66.4
TD 16.2
1-5
EGFP 59.0
TD 11.0
1-6
EGFP 62.9
TD 15.0
2-4
EGFP 59.2
TD 11.4
2-5
EGFP 63.1
TD 12.9
2-6
EGFP 61.1
TD 10.5
3-4
EGFP 56.0
TD 10.2
3-5
EGFP 61.7
TD 10.5
3-6
EGFP 61.7
TD 10.4
SSC-H
BL1-H :: FITC-H
BL2-H :: PE-H

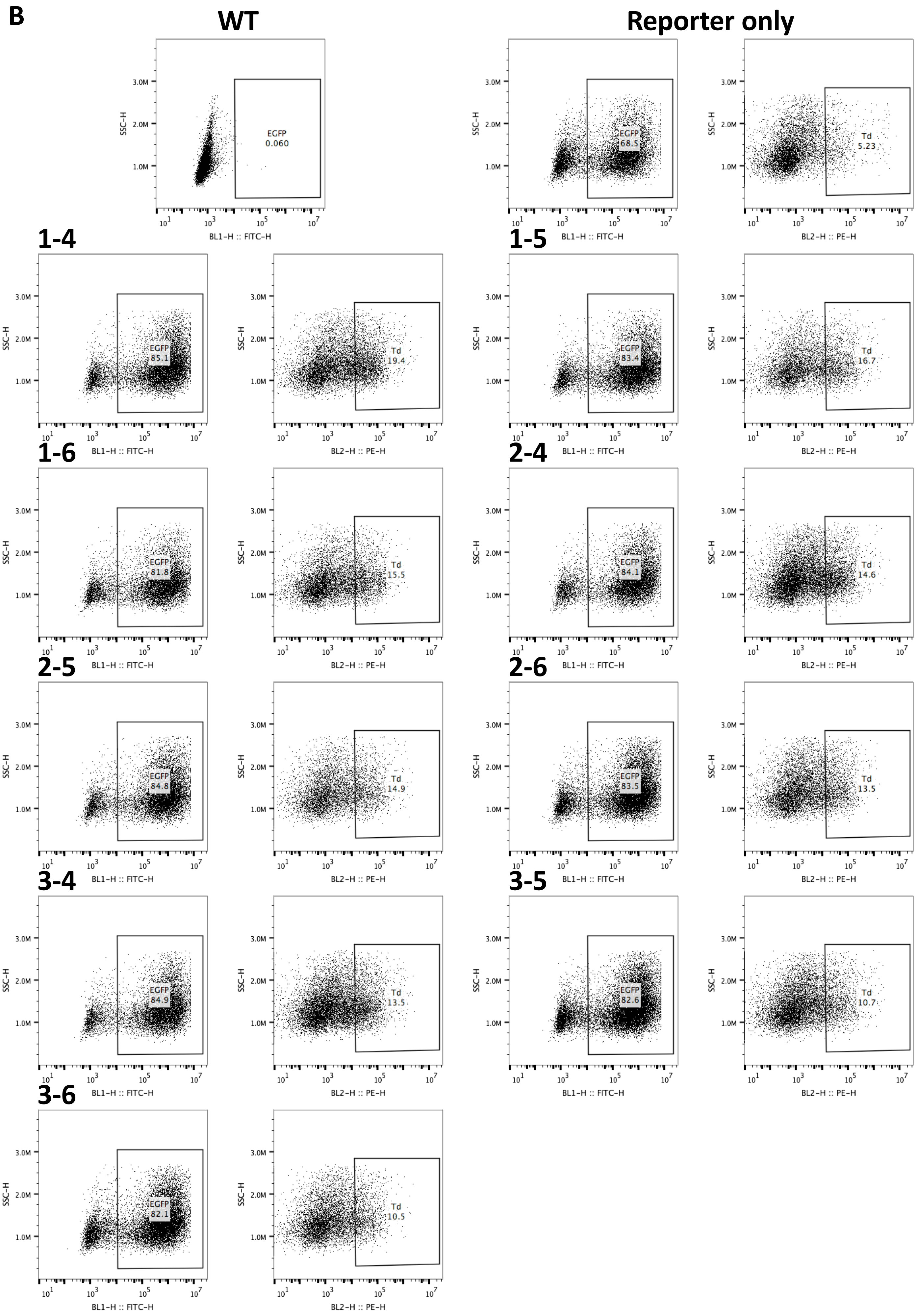

Figure S11. Flow cytometry of HEK293 cells co-transfected with the reporter construct and Cas12a/gRNA.（A,B）2 biological replicates.

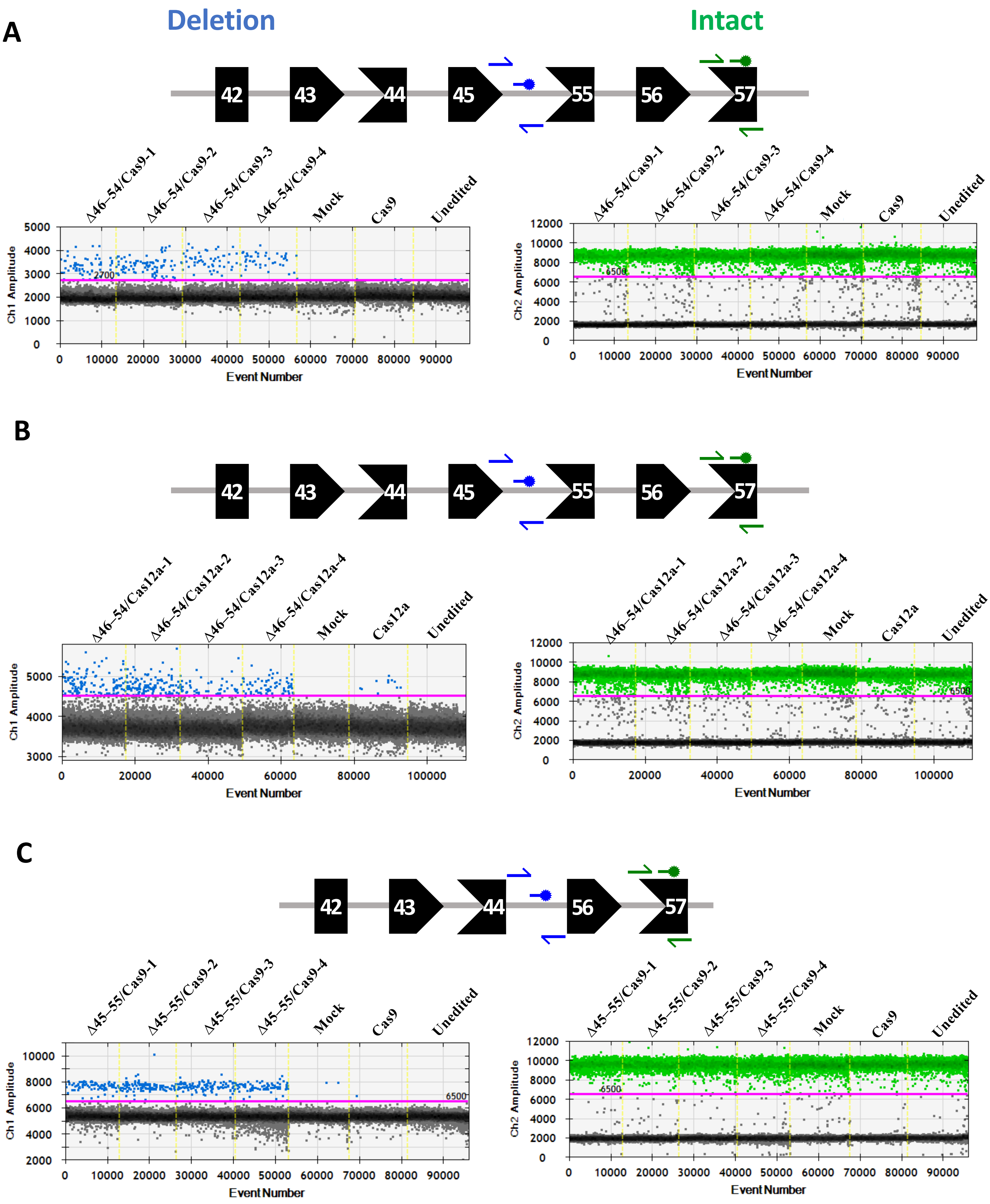

Figure S12.ddPCR analysis for (A) Δ46–54/Cas9 , (B) Δ46–54/Cas12a and (C) Δ45–55/Cas9.

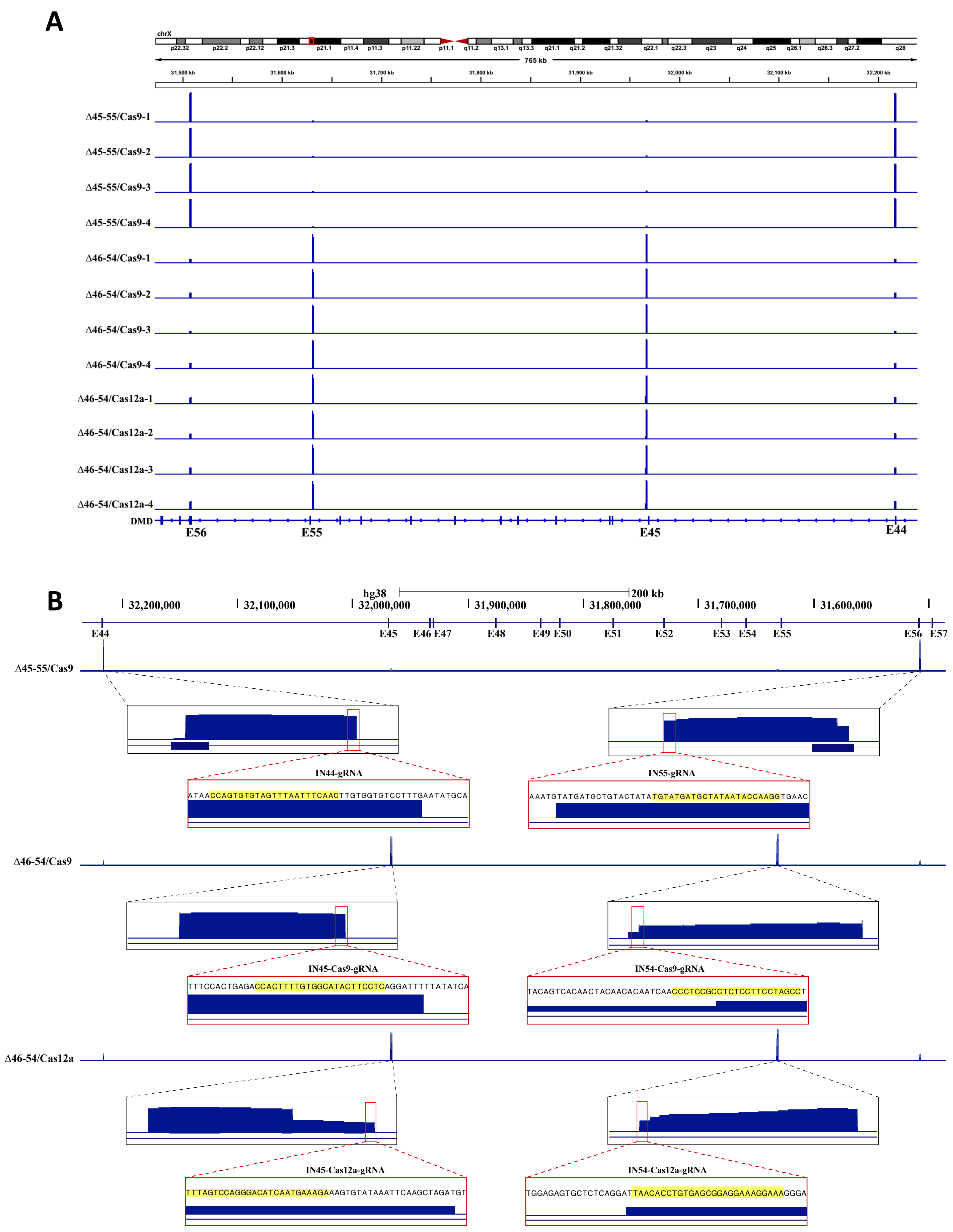

Figure S13 (A) Reads mapped to DMD 44-56 exons regions in the genome were visualized in IGV browser. (B) Replicates shown above were merged together and details of reads coverage and gRNA position in the peak areas was shown. gRNAs were highlighted by yellow color.

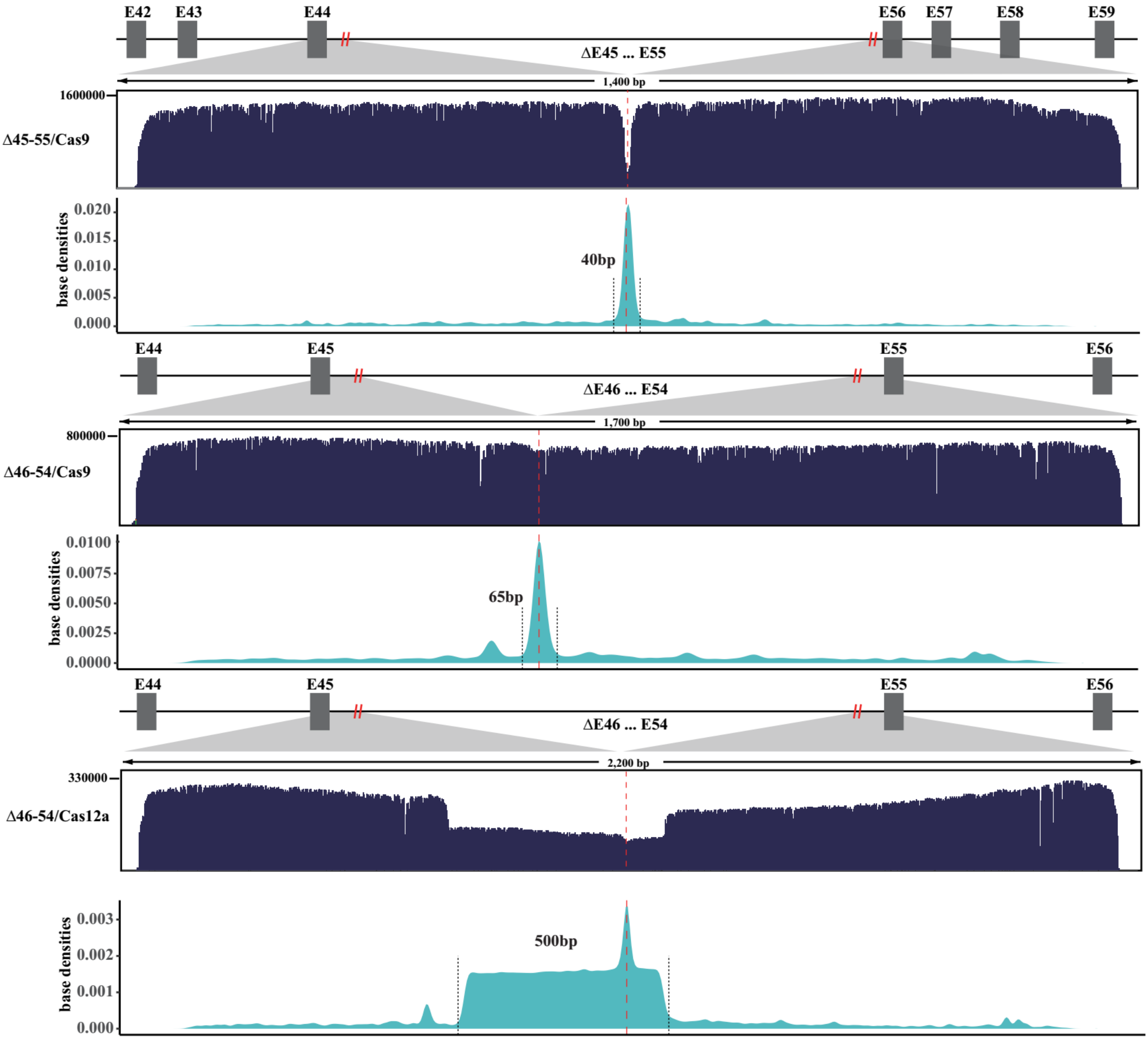

Figure S14. Exploring the mutated base distributions in the Nanopore sequencing regions, and density plots show the mutations regions.

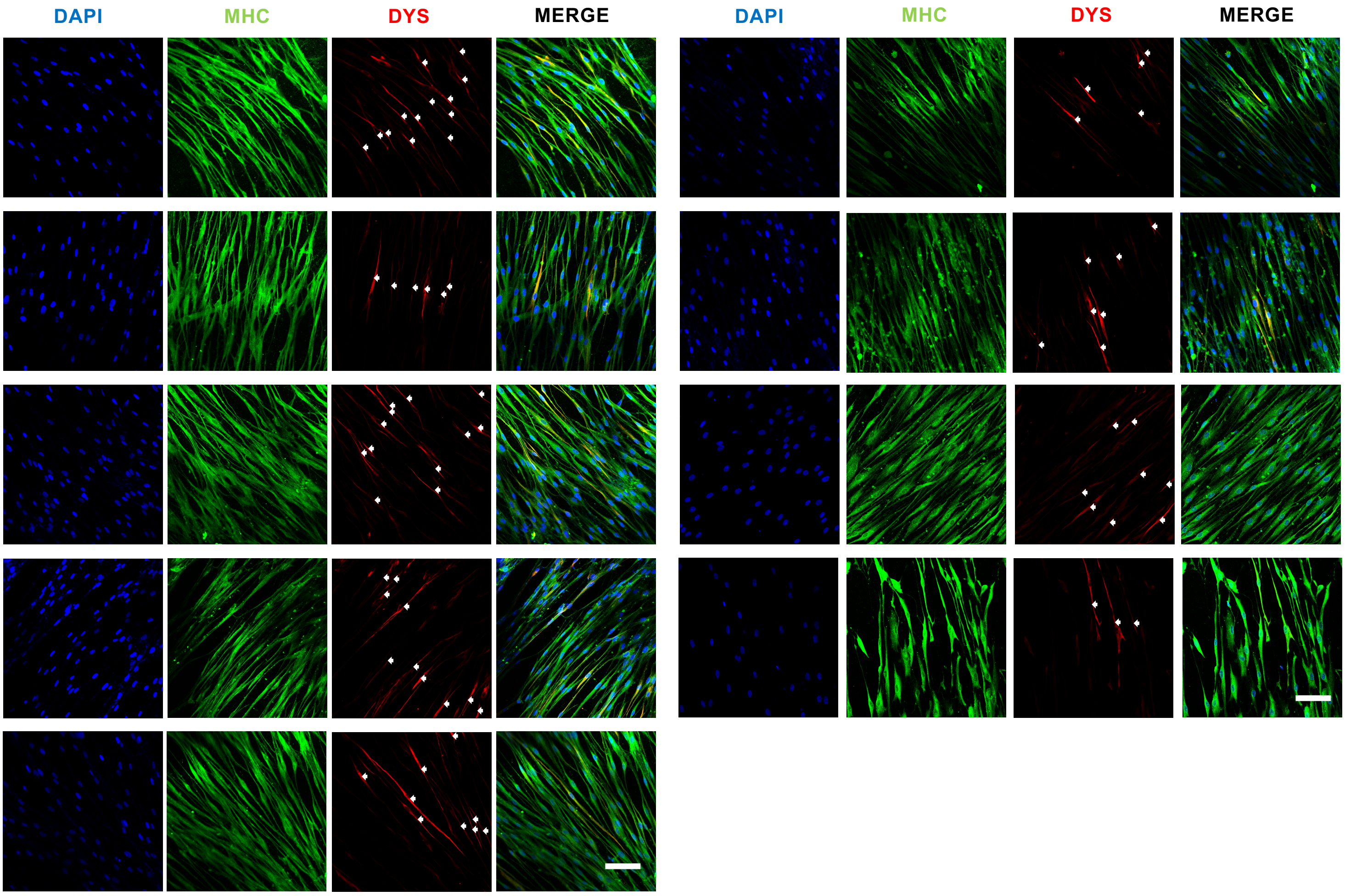

Figure S15. Supplementary images of MHC (green) and dystrophin (red , white arrow) expression in Δ46–54/Cas12a-edited myotubes differentiated from DMD-MDSCs, as determined by immunocytochemistry; nuclei were stained with DAPI (blue). Scale bar, 100 µm.

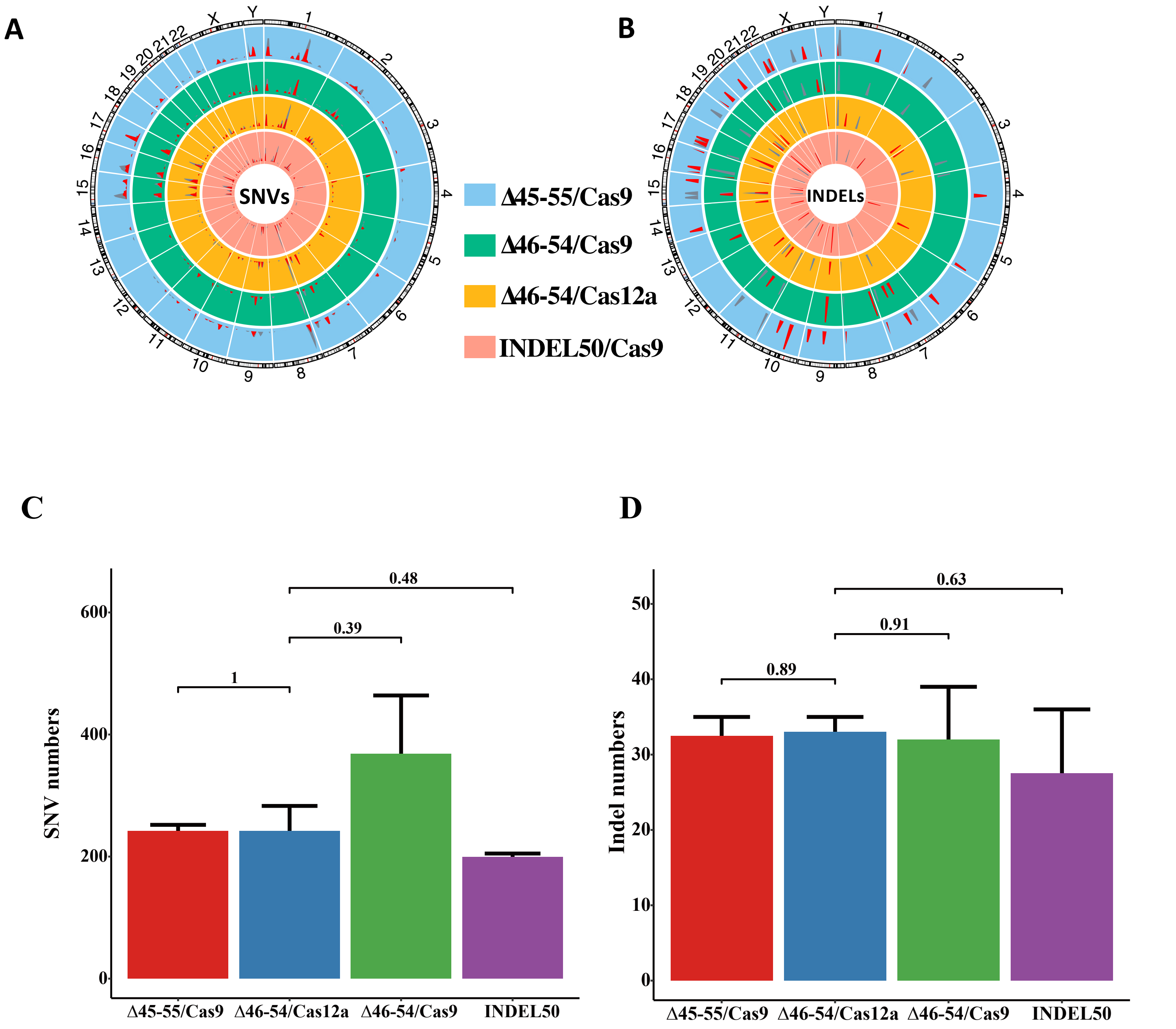

Figure S16. Graph depicting the number of SNVs (A) and indels (B) detected by whole-exome sequencing of Cas9- and Cas12a-targeted cells; Gray corresponds to background and red corresponds to mutation sites, the lengths represent the mutation density in genome, n=2. SNVs (C) and Indels (D) mutation numbers bar plot (n = 2) detected using whole exome sequencing.

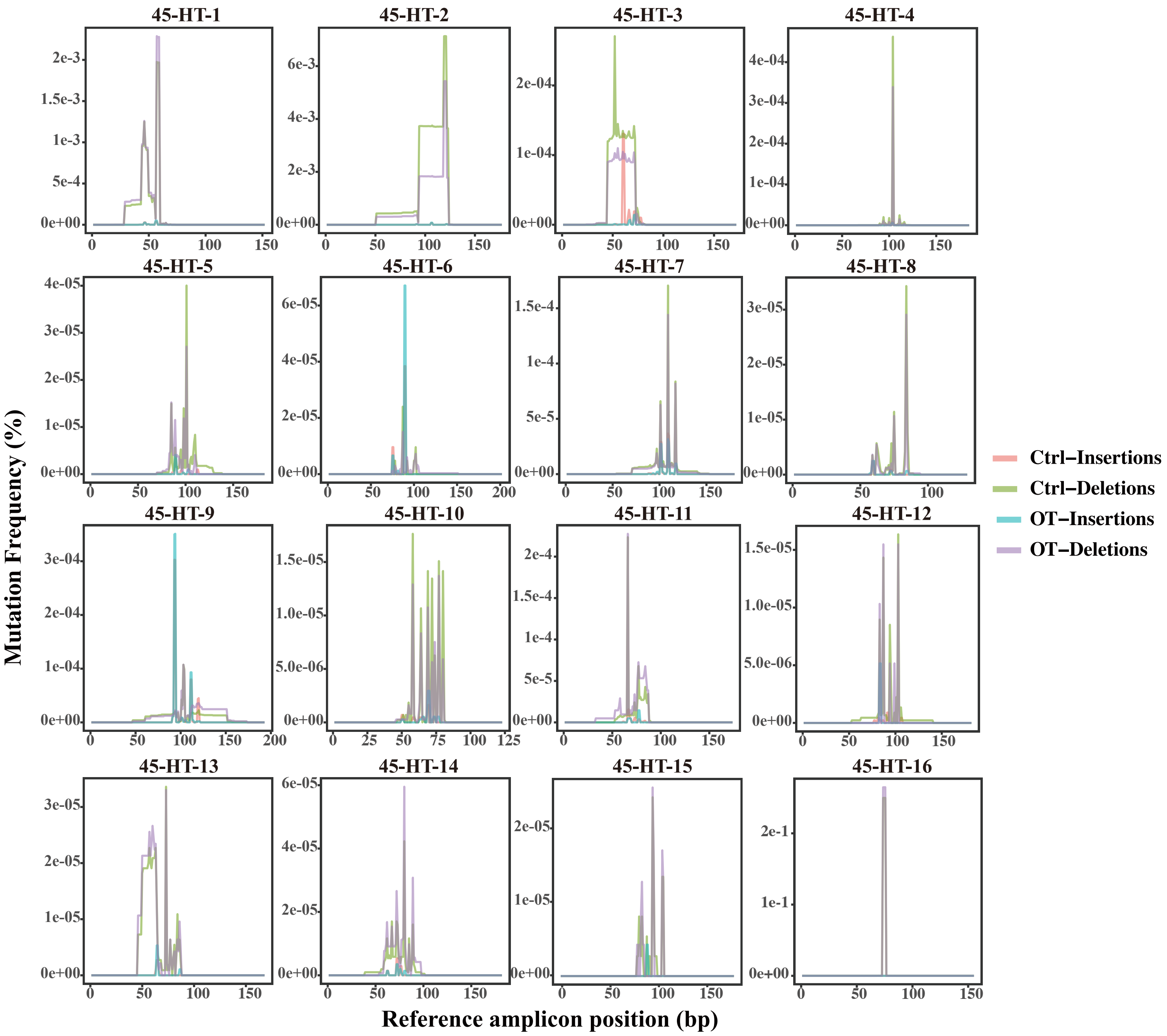

Figure S17. Targeted deep sequencing results for checking the indels from the whole exome sequencing, genomic DNA was from Δ45-55/Cas9 samples. Frequency of insertions and deletions across the entire amplicon was showed in the figure above.

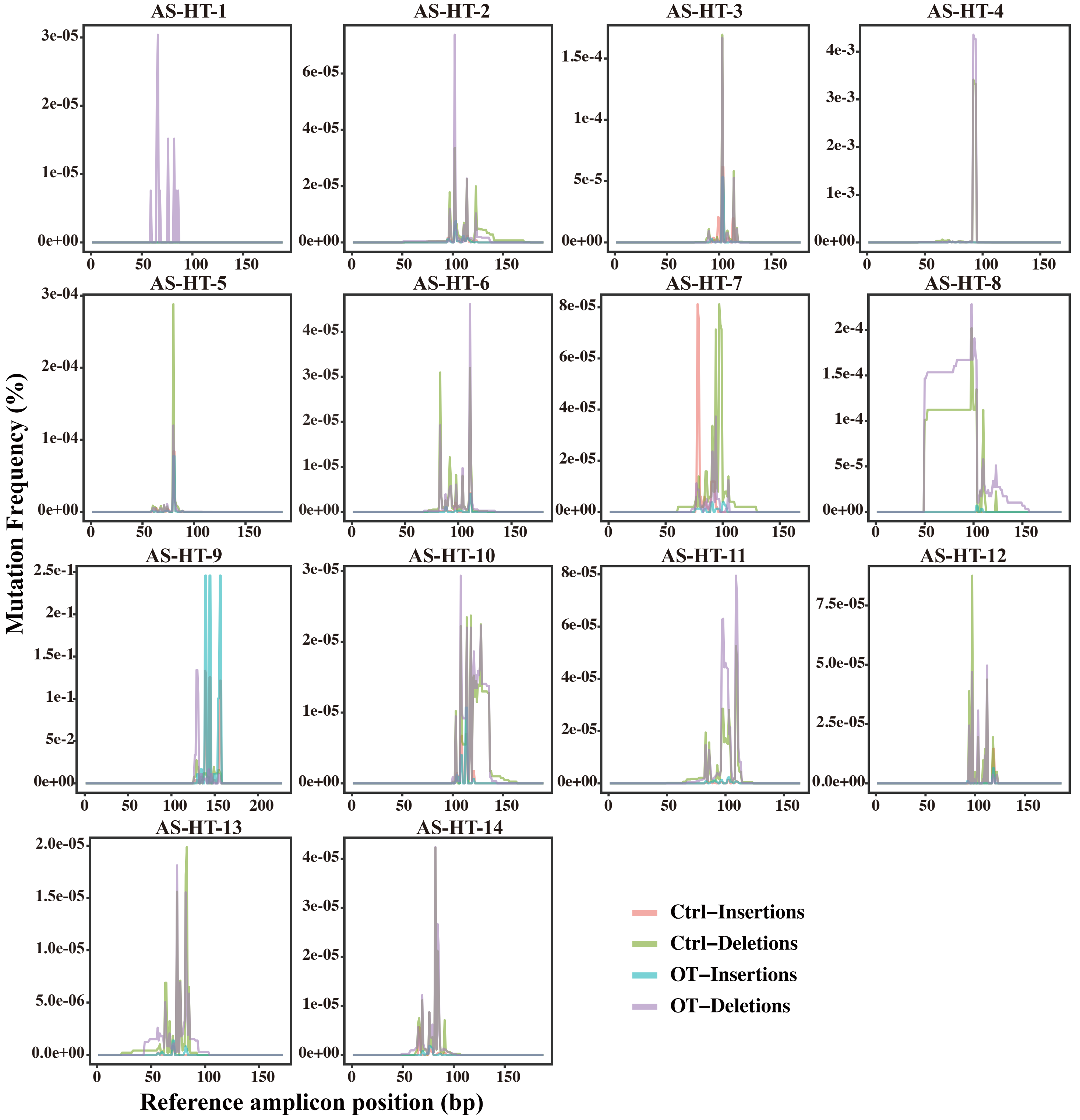

Figure S18. Targeted deep sequencing results for checking the indels from the whole exome sequencing, genomic DNA was from Δ46-54/Cas12a samples. Frequency of insertions and deletions across the entire amplicon was showed in the figure above.

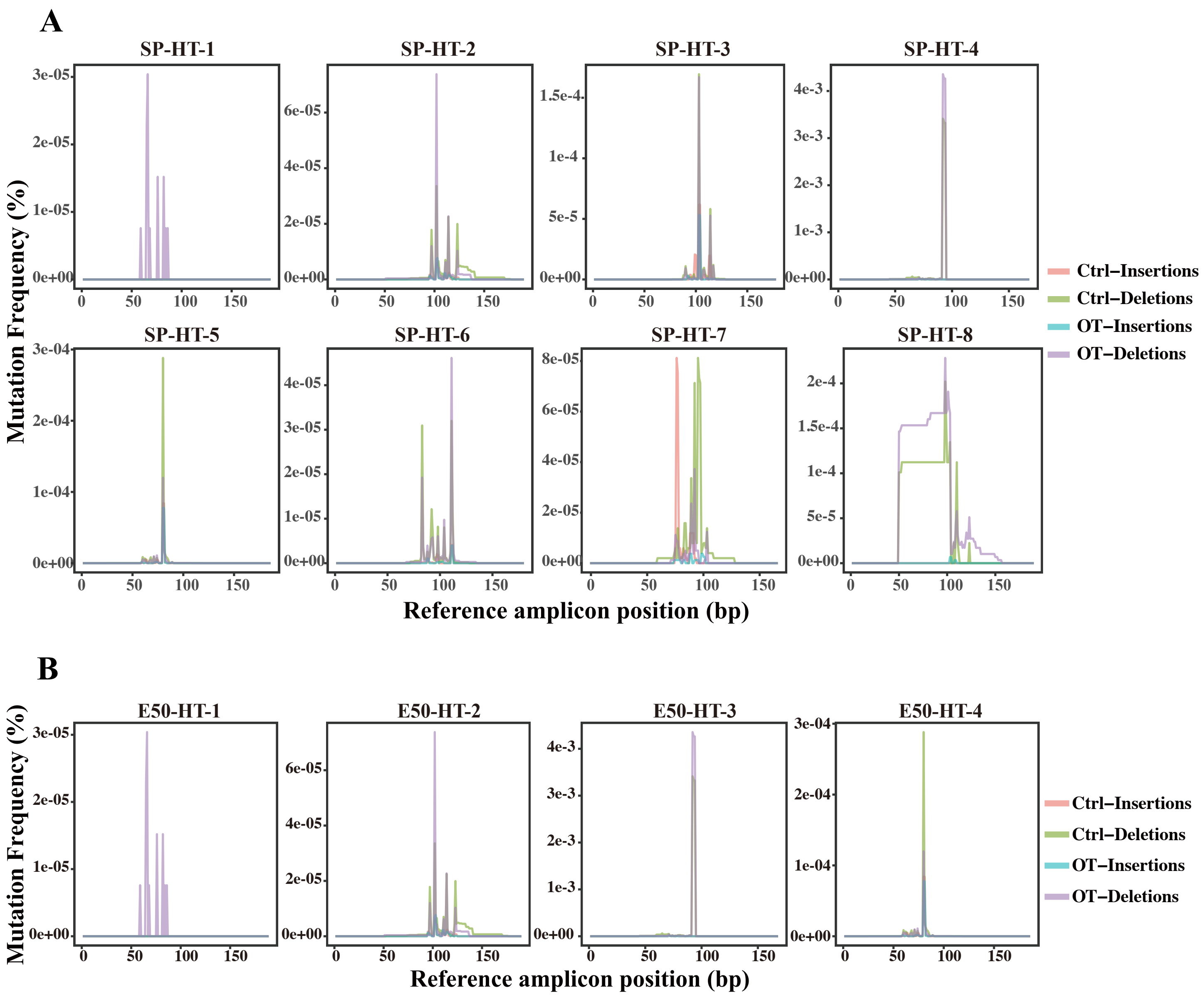

Figure S19. Targeted deep sequencing results for checking the indels from the whole exome sequencing, genomic DNA was from (A) Δ46-54/Cas9 and (B) INDEL50/Cas9 samples. Frequency of insertions and deletions across the entire amplicon was showed in the figure above.

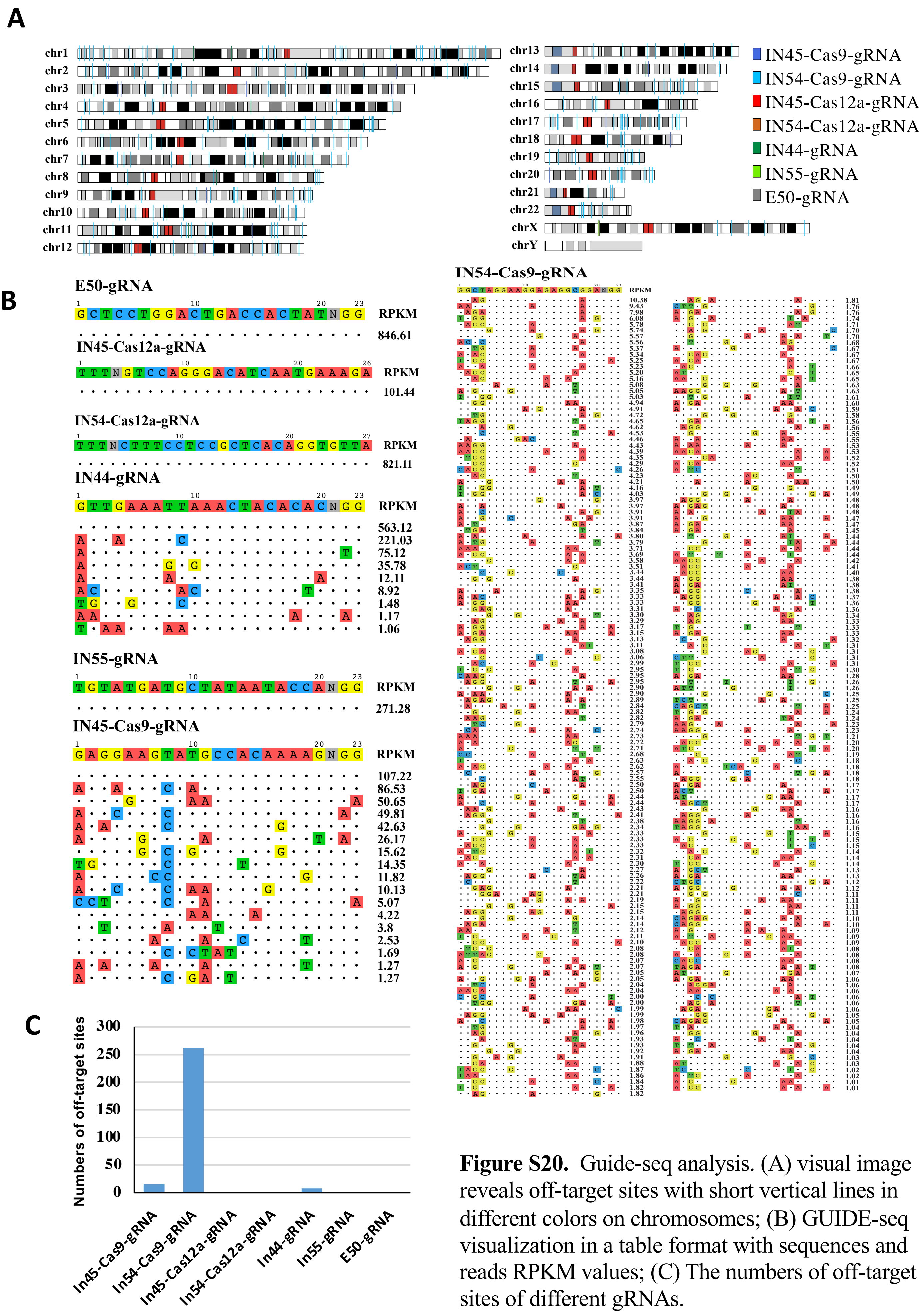

Figure S20. Guide-seq analysis. (A) visual image reveals off-target sites with short vertical lines in different colors on chromosomes; (B) GUIDE-seq visualization in a table format with sequences and reads RPKM values; (C) The numbers of off-target sites of different gRNAs.

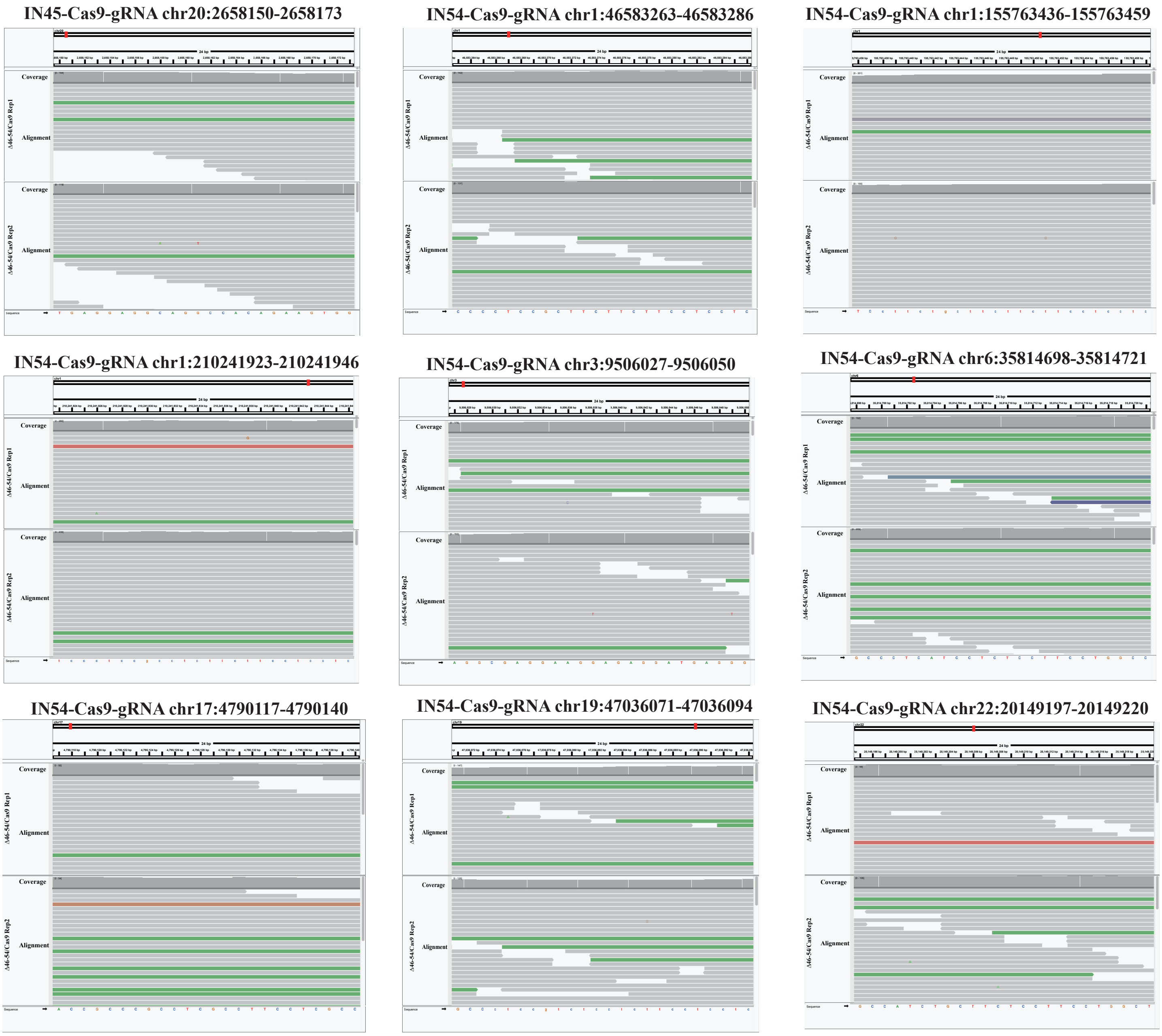

Figure 21. IGV snapshot of regions annotated in exon from GUIDE-seq.
We annotated the results from GUIDE-seq and 9 off-target position was annotated in exon, thus we checked these regions in whole exome sequence aligned bam files in IGV genome browser. The results showed that no mutations appeared in these regions in our whole exome sequence data.

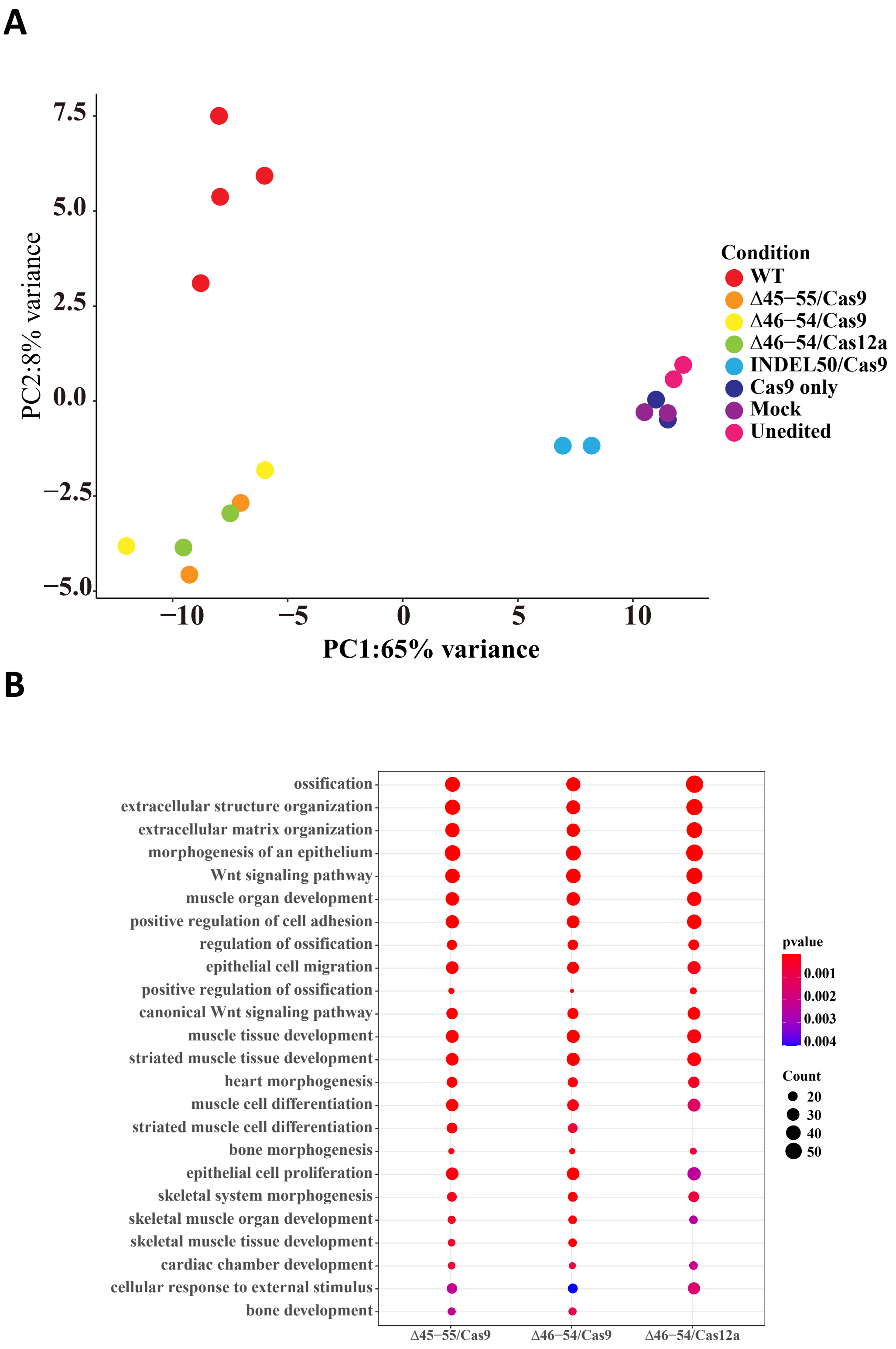

Figure S22. (A) PCA analysis of transcriptome pattern of unedited and edited myotubes differentiated from DMD-MDSCs. (B) Gene Ontology analysis of transcripts in Δ45–55/Cas9, Δ46–54/Cas9 and Δ46–54/Cas12a edited myotubes.

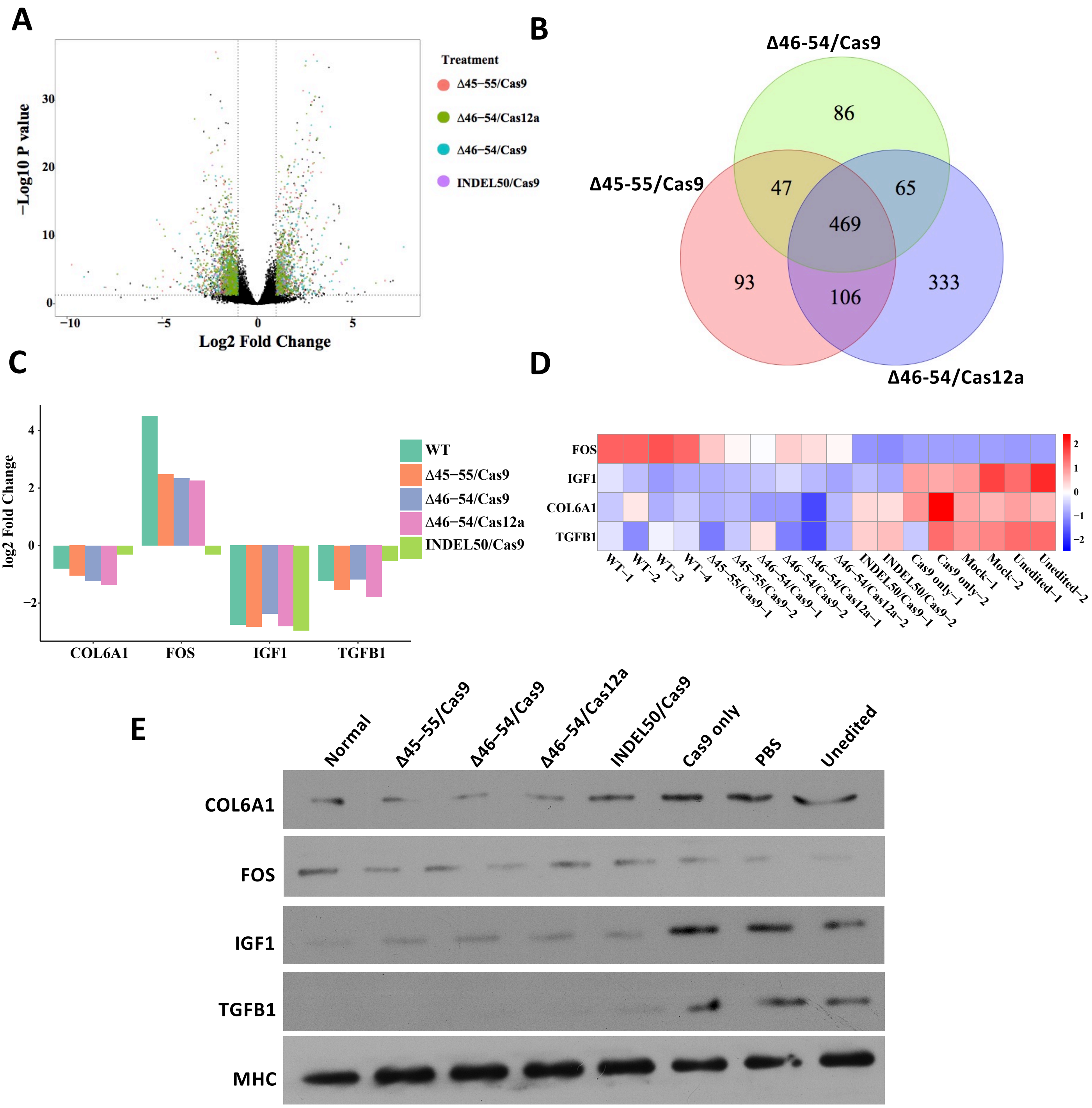

Figure S23. (A) Volcano plot of relative gene expression levels in the unedited and edited myotubes differentiated from DMD-MDSCs. The vertical dotted line delimits down- and up-regulation. In the volcano plot, differential substances(color) and nondifferential substances(black) were determined under the conditions of fold change>2 and false discovery rate-adjusted p value threshold<0.01. (B) Number of genes upregulated and downregulated in the edited myotubes except for INDEL50/Cas9 (Venn diagram). There are 28 commonly differentially expressed genes. (C) bar graph of representative upregulated and downregulated genes related to muscle function of DMD. (D) Hierarchical cluster analysis of representative upregulated and downregulated genes. Red and dark blue represent higher and lower gene expression levels, respectively. (E) Western blot analysis of the upregulated and downregulated genes.

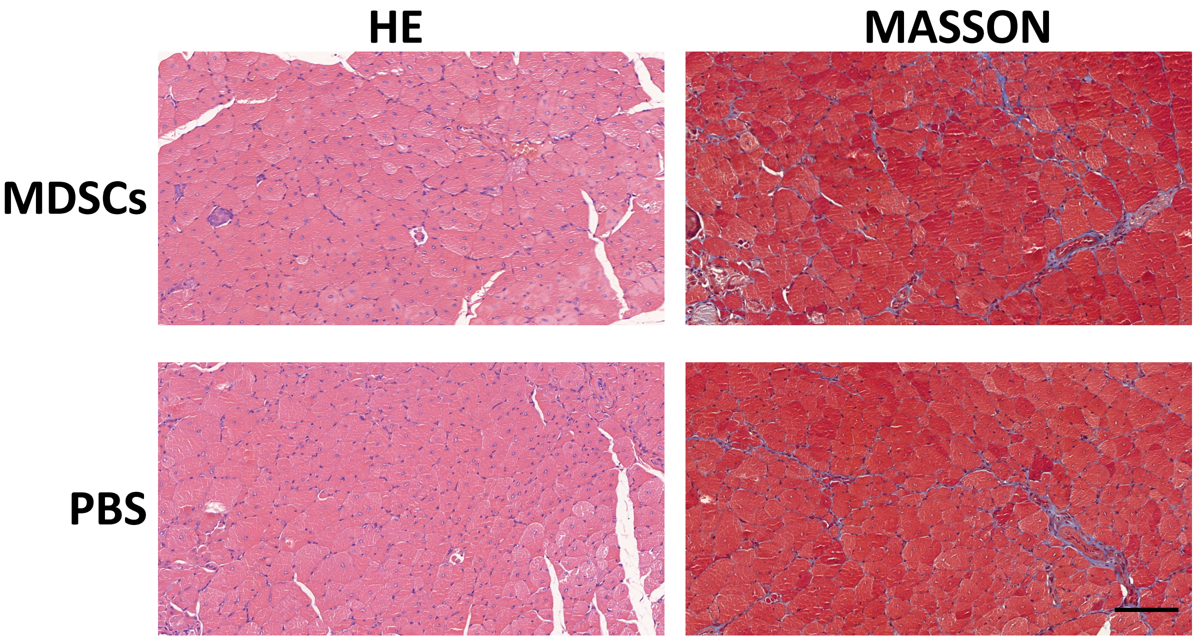

Figure S24. Representative hematoxylin and eosin and Masson's trichrome staining of cardiotoxin-treated TA muscle of NSI mice. Scale bar, 100 mm.

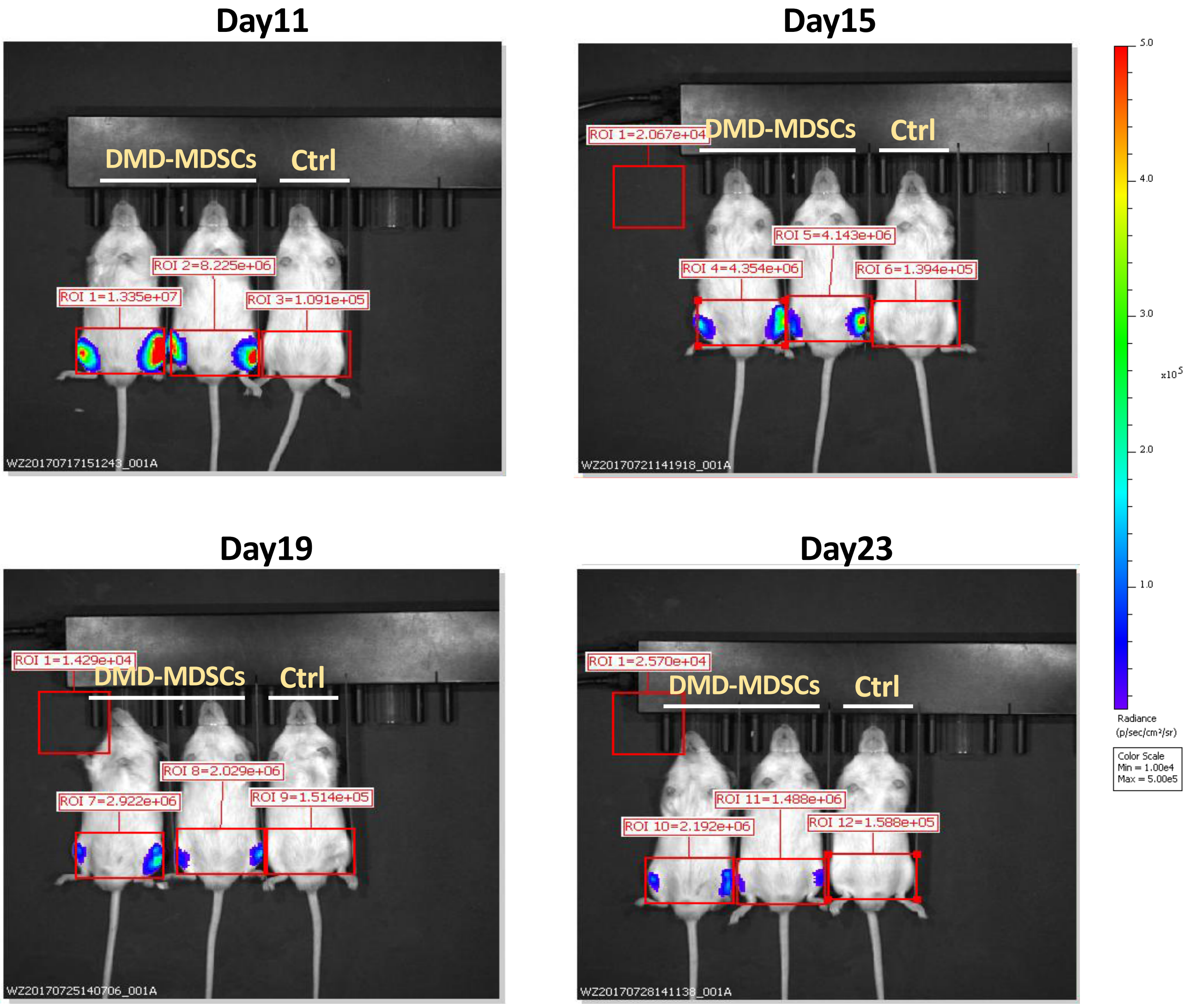

Figure S25. Detailed analysis of bioluminescence for mice shown in Figure6A.

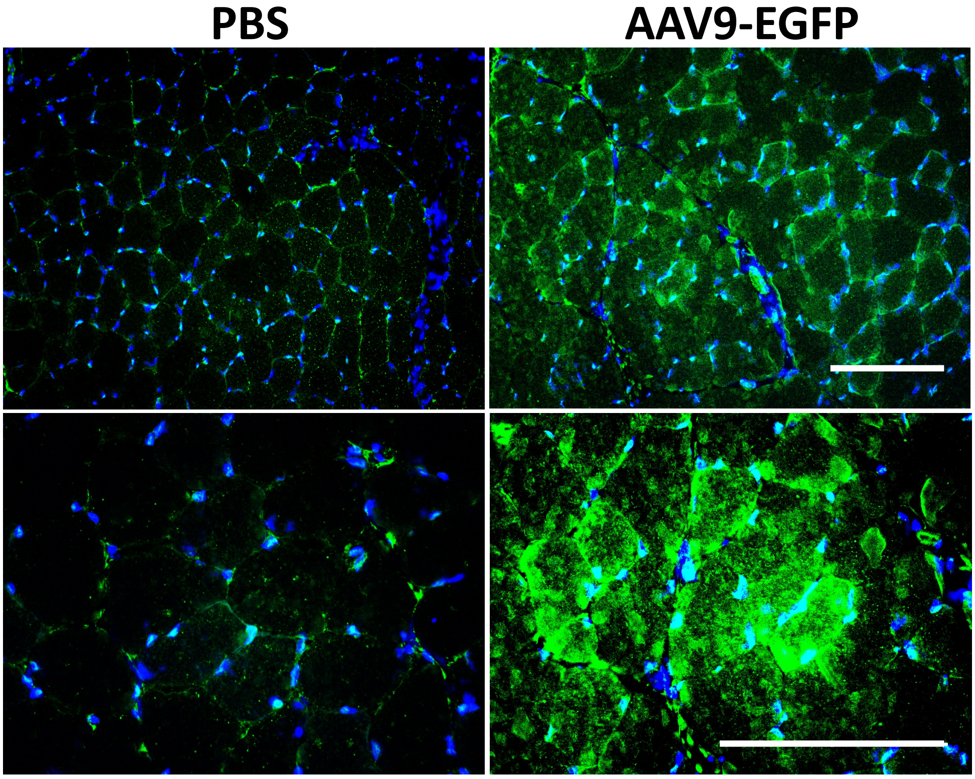

Figure S26. Representative fluorescence image of TA muscle infected with AAV9 expressing EGFP. TA muscle injected with PBS served as a negative control. Scale bar, 100 mm.

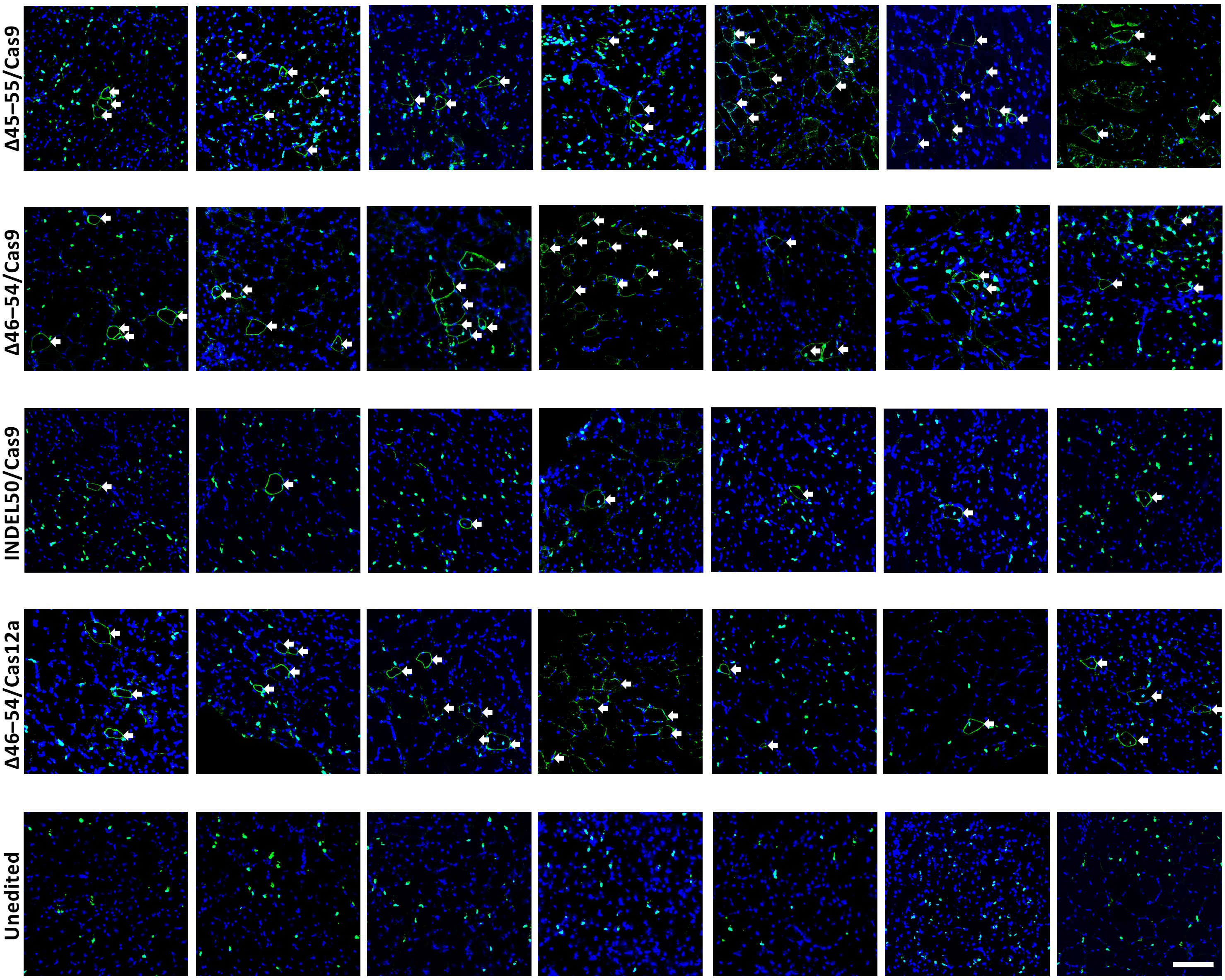

Figure S27. Supplementary Immunofluorescence images of human dystrophin (green signal in the sarcolemma, white arrow) and human lamin A + C (green signal in the nucleus) in the TA muscle of CRISPR-targeted PDX DMD mice; nuclei were counterstained with DAPI (blue). Mice without editing (Unedited) served as the negative control. Scale bar, 100 µm.
